# Neuronal SEL1L-HRD1 ER-associated degradation is essential for motor function and survival in mice

**DOI:** 10.1172/JCI196819

**Published:** 2026-02-19

**Authors:** Mauricio Torres, You Lu, Brent Pederson, Hui Wang, Anna Gretzinger, Liangguang L. Lin, Jiwon Hwang, Xinxin Chen, Alan C. Rupp, Abigail J. Tomlinson, Andrew J. Scott, Zhen Zhao, Daniel R. Wahl, Martin G. Myers, Costas A. Lyssiotis, Ling Qi

**Affiliations:** 1 Department of Molecular Physiology and Biological Physics, University of Virginia School of Medicine, Charlottesville, Virginia, USA.; 2 Department of Molecular & Integrative Physiology and; 3 Division of Metabolism, Endocrinology & Diabetes, Department of Internal Medicine, University of Michigan, Ann Arbor, Michigan, USA.; 4 Department of Radiation Oncology and; 5 Rogel Cancer Center, University of Michigan Medical School, Ann Arbor, Michigan, USA.; 6 Zilkha Neurogenetic Institute, Keck School of Medicine of University of Southern California, Los Angeles, California, USA.; 7 Department of Neurosurgery, University of Michigan Medical School, Ann Arbor, Michigan, USA.; 8 Division of Gastroenterology and Hepatology, Department of Internal Medicine, University of Michigan, Ann Arbor, Michigan, USA.

**Keywords:** Cell biology, Neuroscience, Cell stress, Metabolomics, Mouse models

## Abstract

Hypomorphic variants in the SEL1L-HRD1 ER-associated degradation (ERAD) complex have been linked to severe neurological syndromes in children, including neurodevelopmental delay, intellectual disability, motor dysfunction, and early death. Despite this association, its physiological importance and underlying mechanisms in neurons remain poorly understood. Here, we show that neuronal SEL1L-HRD1 ERAD is essential for maintaining one-carbon metabolism, motor function, and overall viability. Neuron-specific deletion of *Sel1L* in mice (*Sel1L^SynCre^*) resulted in growth retardation, severe motor impairments, and early mortality by 9 weeks of age — mirroring core clinical features observed in affected patients — despite preserved neuronal numbers and only modest ER stress. Multiomics analyses, including single-nucleus RNA sequencing and metabolomics, revealed significant dysregulation of one-carbon metabolism in ERAD-deficient brains. This included activation of the serine, folate, and methionine pathways, accompanied by elevated levels of *S*-adenosylmethionine and related metabolites, likely resulting from induction of the integrated stress response. Together, these findings uncover a previously unappreciated role for neuronal SEL1L-HRD1 ERAD in coordinating ER protein quality control with metabolic adaptation, providing insight into the molecular basis of ERAD-related neurodevelopmental disease.

## Abstract 2

Using a neuron-specific *Sel1L* (vs. *Ire1a*) knockout mouse model, we demonstrate that *Sel1L* deficiency, not *Ire1a* deficiency, activates integrated stress responses, rewires one-carbon metabolism, and impairs motor function and survival.

## Graphical abstract

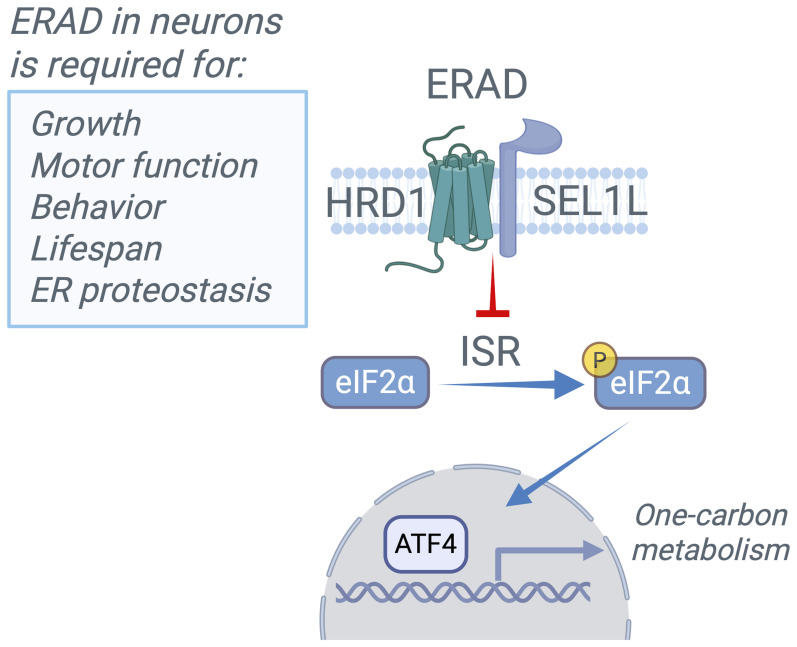

## Introduction

Protein synthesis and folding within the endoplasmic reticulum (ER) are tightly coordinated processes essential for cellular homeostasis (1–3). To maintain proteostasis and prevent the accumulation of aberrant proteins, the ER relies on quality control mechanisms, most notably the unfolded protein response (UPR) and ER-associated degradation (ERAD) pathways (4). ERAD targets misfolded or unassembled proteins for retrotranslocation to the cytosol and subsequent proteasomal degradation (5–8). Among ERAD pathways, the SEL1L-HRD1 complex is the most evolutionarily conserved, from yeast to mammals (9–11). HRD1, a multipass transmembrane E3 ubiquitin ligase, forms the retrotranslocation channel (8, 9, 12), while its adaptor, SEL1L, ensures HRD1 stability and substrate recognition (13–18). Deletion of Sel1L or Hrd1 — either globally or in an inducible fashion — results in embryonic or early postnatal lethality, underscoring their essential roles in development and tissue homeostasis (16, 19–21). Cell type–specific knockout studies have further revealed the importance of SEL1L-HRD1 ERAD in diverse physiological contexts, including lipid and glucose metabolism, immune regulation, thermogenesis, and organ integrity (6, 7, 22–25). Recent work from our group has identified pathogenic variants in SEL1L and HRD1 in pediatric patients presenting with neurodevelopmental delay, intellectual disability, motor dysfunction, and early mortality — clinical features that define a syndrome we refer to as ERAD-associated neurodevelopmental disorder with onset in infancy (ENDI) (26–28).

Neurons, as highly specialized and long-lived postmitotic cells, are particularly dependent on efficient protein folding and ER quality control for lifelong function. The ER is central to the folding and maturation of numerous membrane and secretory proteins vital for neurotransmission, synaptic plasticity, and cellular viability (29, 30). Despite the importance of proteostasis in neurons, the functional role of ERAD in the nervous system remains largely unclear. Elucidating ERAD’s role in neurons is critical not only for understanding the pathogenesis of ENDI but also for exploring broader implications in neurodegenerative diseases and age-associated cognitive decline. Prior studies have shown that deletion of Sel1L in hypothalamic neurons disrupts the maturation of prohormones and receptors — including pro–arginine vasopressin, pro-opiomelanocortin, and the leptin receptor — leading to altered fluid balance and energy homeostasis (31–34). Similarly, selective loss of Sel1L in cerebellar Purkinje cells causes early-onset ataxia and neuron degeneration, supporting a cell-autonomous role for ERAD in neuronal integrity (35). However, the global consequences of neuronal ERAD deficiency in vivo remain poorly defined.

In this study, we generated neuron-specific conditional knockout models targeting SEL1L or the UPR sensor IRE1α to dissect the importance of SEL1L-HRD1 ERAD to brain function. We found that deletion of *Sel1L*, but not *Ire1a*, in a subset of neurons led to growth retardation, motor deficits, and early mortality. Remarkably, these phenotypes occurred in the absence of overt neuronal loss or robust UPR activation. Multiomics analyses, including single-nucleus RNA sequencing (snRNA-seq) and metabolomics, revealed broad upregulation of one-carbon metabolism, a pathway central to nucleotide biosynthesis and methylation reactions essential for brain development (36). Disruption of one-carbon metabolism has been implicated in multiple neurodevelopmental and neuropsychiatric disorders, including neural tube defects, autism spectrum disorder, and schizophrenia, often through impaired methylation, altered neurotransmitter synthesis, and elevated homocysteine levels (36–38). Furthermore, the observed metabolic shifts were accompanied by activation of the integrated stress response (ISR) (39), suggesting that ERAD deficiency may lead to metabolic reprogramming.

## Results

### SEL1L and HRD1 are highly enriched in neurons.

**Figure 1:**
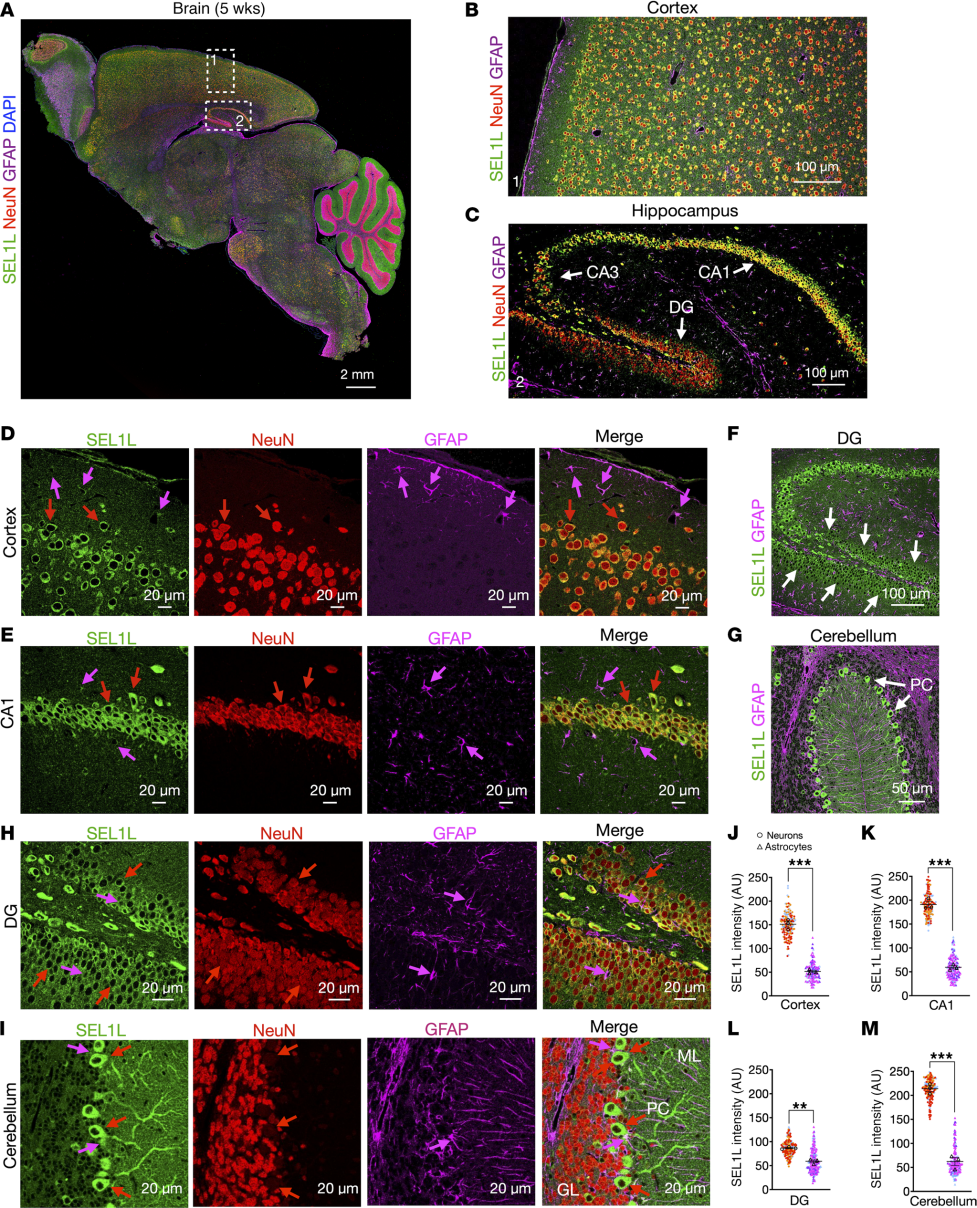
SEL1L protein is highly expressed in neurons. (**A**) Representative confocal image of SEL1L (green), NeuN (red), glial fibrillary acidic protein (GFAP) (magenta), and DAPI (blue) staining in a sagittal brain section of a 5-week-old mouse. The magnifications of the selected regions for cortex and hippocampus, are shown in panels **B** and **C**, respectively. White arrows in panel **C** indicate the Cornu Ammonis 1 (CA1), CA3, and dentate gyrus (DG) regions of the hippocampus. (**D** and **E**) Representative zoomed-in images for cortex (**D**) and CA1 (**E**) regions showing separated channels for SEL1L, NeuN, and GFAP staining. Red and magenta arrows indicate the signal for neurons and astrocytes, respectively. (**F** and **G**) Magnification of the DG (**F**) and cerebellum (**G**) showing the localization of SEL1L and GFAP. White arrows indicate SEL1L-positive signal in granule cells (**F**) and Purkinje cells (PC) (**G**). (**H** and **I**) Representative zoomed-in images for DG (**H**) and cerebellum (**I**) showing separated channels for SEL1L, NeuN, and GFAP staining. The molecular layer (ML) and granular layer (GL) are indicated as reference points in panel **I**. Arrows, neurons (red) and astrocytes (magenta). (**J**–**M**) Quantitation of SEL1L signal intensity in neurons (red) and astrocytes (magenta) in cortex (**J**), CA1 (**K**), DG (**L**), and cerebellum (**M**) regions of 5-week-old mice (total of 180–200 cells from 3 mice per group; data from each mouse are shown in different colors). Data are shown as the mean ± SEM. **P < 0.01, and ***P < 0.001 by 2-tailed Student’s *t* test (**J**–**M**). Scale bar: 2 mm (**A**); 100 μm (**B**, **C**, and **F**); 50 μm (**G**); 20 μm (**D**, **E**, **H**, and **I**).

To assess the cell type–specific distribution of ERAD components in the brain — including neurons and glial cells such as astrocytes, oligodendrocytes, and microglia (40) — we examined SEL1L and HRD1 protein expression in coronal sections from 5-week-old wild-type (WT) mice by immunofluorescence (Figure 1 and Supplemental Figure 1; supplemental material available online with this article; https://doi.org/10.1172/JCI196819DS1). Neurons were identified by NeuN, a nuclear and perinuclear marker of mature neurons, while astrocytes, the most abundant glia cell in brain, were labeled with glial fibrillary acidic protein (GFAP). In the cerebral cortex, SEL1L was predominantly localized to the perinuclear region of NeuN-positive neurons, consistent with its localization to the ER (Figure 1, A, B, and D). In contrast, astrocytes — distinguished by their smaller, stellate morphology — exhibited relatively weak SEL1L expression (Figure 1, A, B, D, and J). In the hippocampus, SEL1L was strongly expressed in neuronal layers of Cornu Ammonis (CA) 1 and CA3, as well as the dentate gyrus (DG), whereas astrocytes exhibited markedly weaker signals (Figure 1, C, E, F, H, K, and L). Similarly, in the cerebellum, SEL1L expression was notably higher in Purkinje neurons compared with surrounding glial cells (Figure 1, G, I, and M). HRD1 exhibited a comparable distribution pattern and subcellular localization across these brain regions (Supplemental Figure 1). Collectively, these data demonstrate that the SEL1L-HRD1 ERAD complex is highly enriched in neurons across multiple brain regions, supporting a potential role in neuronal function and maintenance.

### Generation and validation of a neuron-specific Sel1L-deficient mouse model.

**Figure 2:**
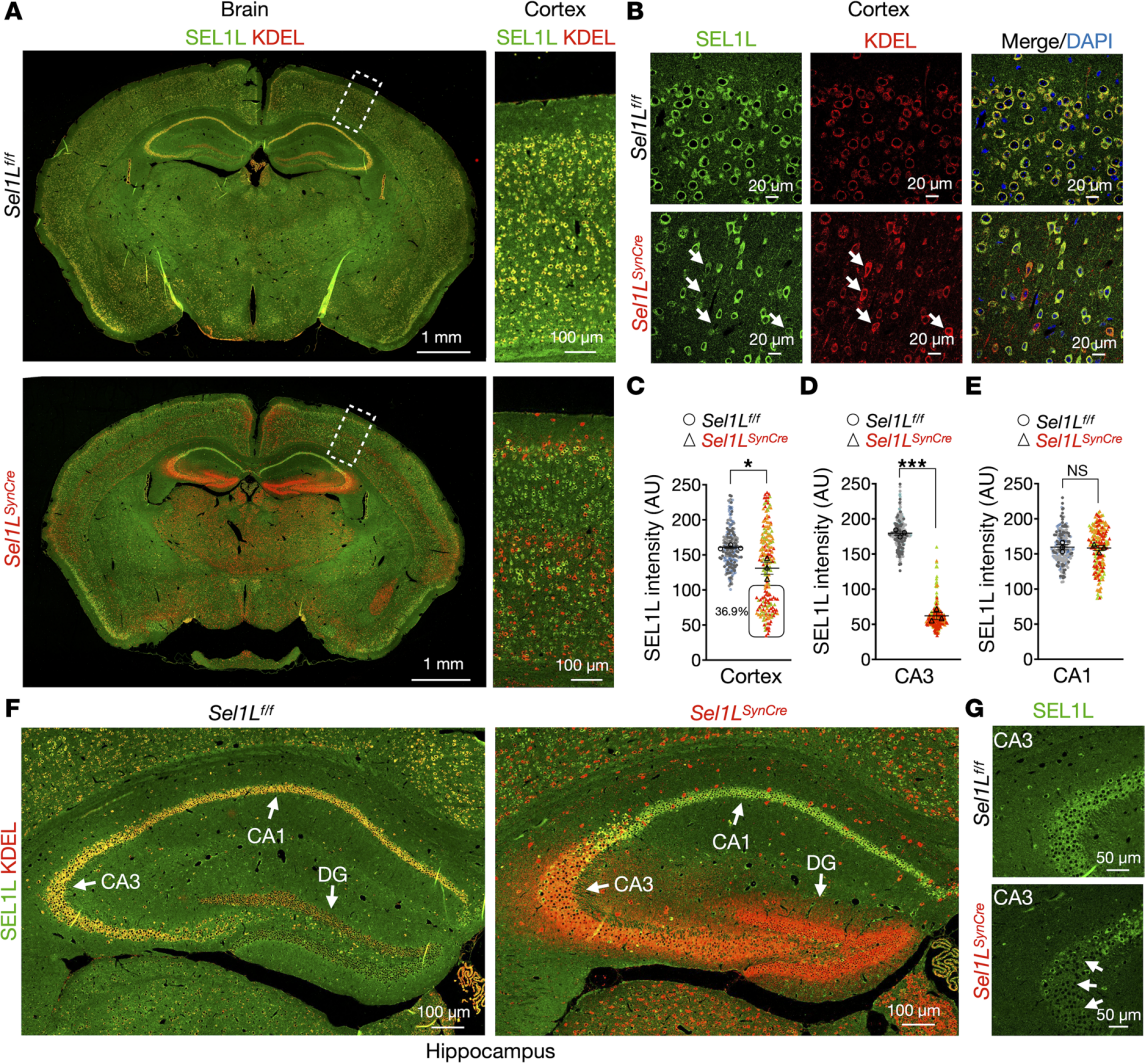
Generation of a neuronal *Sel1L*-deficient mouse model. (**A**) Representative confocal images of SEL1L (green) and KDEL (red) staining in coronal brain sections of 5-week-old wild-type (*Sel1L^fl/fl^*, upper panel) and *Sel1L*-deficient mice (*Sel1L*^SynCre^). The magnification of the selected region for cerebral cortex is showed in the lateral panel for each image. (**B**) Representative zoom-in images for the external layer of the cortex showing separated channels for SEL1L and KDEL staining. White arrows indicate cells with reduced levels of *Sel1L*. Elevated KDEL signal is used to identify *Sel1L*-deficient neurons. (**C**–**E**) Quantitation of SEL1L signal intensity in cortex (**C**), CA3 (**D**), and CA1 (**E**) regions of 5-week-old mice (total of 180–200 cells from 3 mice per group; data from each mouse are shown in different colors). (**F**) Magnification of the hippocampus for wild-type (left panel) and *Sel1L*-deficient mice (right panel). White arrows indicate the CA1, CA3, and DG regions of the hippocampus. (**G**) High-magnification images showing the reduction of SEL1L signal in the CA3 region of the hippocampus (white arrows). Data are shown as the mean ± SEM. *P < 0.05, and ***P < 0.001 by 2-tailed *t* test. NS; not significant. (**C**–**E**). Scale bar: 1 mm (**A**); 100 μm (**A** lateral panel, **F**); 50 μm (**G**); 20 μm (**B**).

To investigate the role of the SEL1L-HRD1 ERAD pathway in neurons, we generated *Sel1L^SynCre^* mice by crossing *Sel1L*-floxed (*Sel1L^fl/fl^*) animals with a Synapsin I promoter–driven Cre line (41). The Synapsin I promoter becomes active around embryonic day 14–16, depending on the brain region, and remains active in postmitotic neurons (41). To assess SEL1L deletion in the brain, we performed confocal microscopy following immunofluorescence staining for SEL1L and the ER marker KDEL on coronal sections from 5-week-old mice (Figure 2). KDEL signal is known to increase upon ERAD deficiency as part of a compensatory response (42). In WT *Sel1L^fl/fl^* littermates, SEL1L colocalized with KDEL throughout the cortex and hippocampus (yellow, Figure 2, A, B, and F). In contrast, *Sel1L^SynCre^* mice showed loss of SEL1L in subsets of neurons, accompanied by a marked increase in KDEL staining in both regions (red, Figure 2, A, B, and F). These regions are critical for motor function, learning, and memory (43, 44). Quantification revealed that approximately 37% of cortical neurons lacked detectable SEL1L (Figure 2C), while deletion efficiency approached 100% in the CA3 and DG subregions of the hippocampus (arrows, Figure 2, D, F, and G). Interestingly, SEL1L expression in the CA1 region was largely preserved (Figure 2, E and F). Western blot analysis confirmed reduced SEL1L levels in both cortex and hippocampus (Supplemental Figure 2). HRD1 protein was also decreased in the hippocampus but remained unchanged in the cortex. In contrast, OS9, a lectin and known ERAD substrate, was significantly upregulated in both regions (Supplemental Figure 2, A–D), indicating ERAD dysfunction. Together, these results validate the successful generation of a neuron-specific *Sel1L*-knockout model, with regionally distinct deletion efficiencies and evidence of ERAD disruption in affected neurons.

### Neuronal deficiency of SEL1L, but not IRE1α, leads to growth retardation and premature lethality.

**Figure 3:**
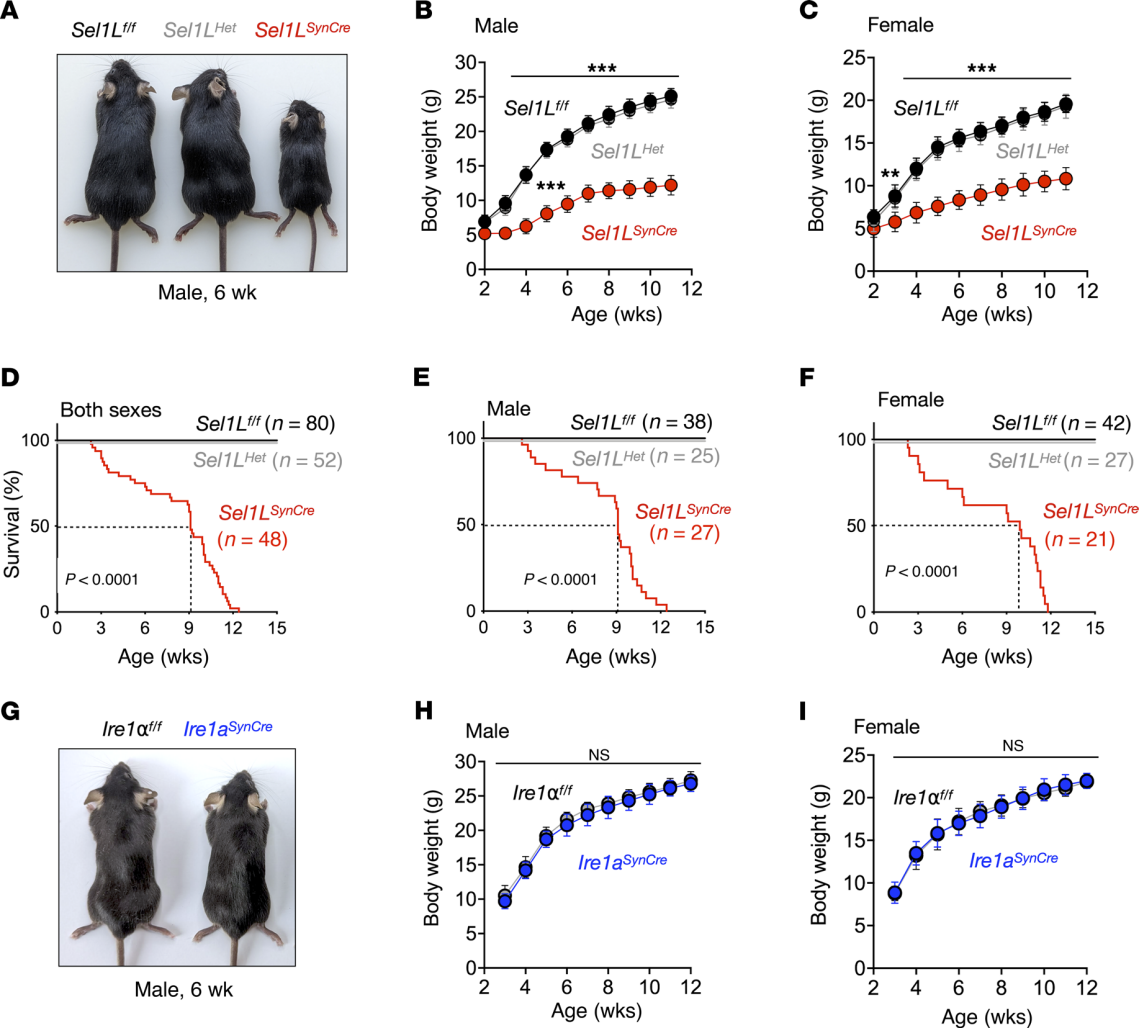
Growth retardation and premature lethality of *Sel1L^SynCre^* mice but not *Ire1a*^SynCre^ mice. (**A**) Representative images of 6-week-old wild-type (*Sel1L^fl/fl^*), heterozygote (*Sel1L^Het^*), and neuronal *Sel1L*-deficient (*Sel1L^SynCre^*) male mice. (**B** and **C**) Growth curve for male (**B**) and female (**C**) *Sel1L^fl/fl^*, *Sel1L^Het^*, and *Sel1L^SynCre^* mice (2–11 weeks, *n* = 12–16 mice per group). (**D**–**F**) Kaplan-Meier mouse survival curves of both sexes (**D**), males (**E**), and females (**F**). Overall survival was followed up for 15 weeks. The number of animals is indicated for each genotype. *P* value was calculated using the log-rank test. (**G**) Representative images of 6-week-old wild-type (*Ire1a^fl/fl^*) and neuronal *Ire1a-*deficient (*Ire1a^SynCre^*) male mice. (**H** and **I**) Growth curve for male (**H**) and female (**I**) *Ire1a^fl/fl^* and *Ire1a^SynCre^* mice (3–12 weeks, *n* = 12–14 mice per group). Body weight data are presented as mean ± SEM and were analyzed by two-way ANOVA. **P < 0.01; ***P < 0.001; NS, not significant.

*Sel1L^SynCre^* mice were born at Mendelian ratios and were phenotypically indistinguishable from their littermate controls at birth. However, by 2 weeks of age, both male and female *Sel1L^SynCre^* mice exhibited pronounced growth retardation, with body weights reaching only approximately 50% of those of WT littermates by 2 months of age (Figure 3, A–C). These mice also demonstrated increased postweaning mortality, with a median survival of 9 weeks (Figure 3, D–F). In terminal stages, *Sel1L^SynCre^* mice developed tremors and typically died before 12 weeks of age (Supplemental Video 1). Histopathological analysis of peripheral tissues — including brown and white adipose tissue, liver, skeletal muscle, kidney, and pancreas — revealed no overt abnormalities (Supplemental Figure 3). To determine whether disruption of the UPR elicits similar phenotypes, we generated *Ire1a^SynCre^* mice, in which IRE1α, a major UPR sensor (45), was conditionally deleted in neurons using the same Synapsin I promoter–driven Cre line. Western blotting confirmed efficient deletion of IRE1α in both cortex and hippocampus (Supplemental Figure 4A). In contrast with *Sel1L^SynCre^* mice, *Ire1a^SynCre^* mice developed normally and were indistinguishable from their WT littermates with respect to body weight, appearance, and overall health (Figure 3, G–I). Moreover, binding immunoglobulin protein (BiP) expression, PKR-like ER kinase (PERK) levels, and eukaryotic initiation factor 2 alpha (eIF2α) phosphorylation remained unaltered, indicating preserved ER homeostasis (Supplemental Figure 4, A and B). These findings establish that the SEL1L-HRD1 ERAD pathway, but not neuronal IRE1α signaling, is essential for maintaining neuronal homeostasis and is critically required for postnatal growth and survival.

### Impaired motor activity and behavioral abnormalities in Sel1L^SynCre^ mice.

**Figure 4:**
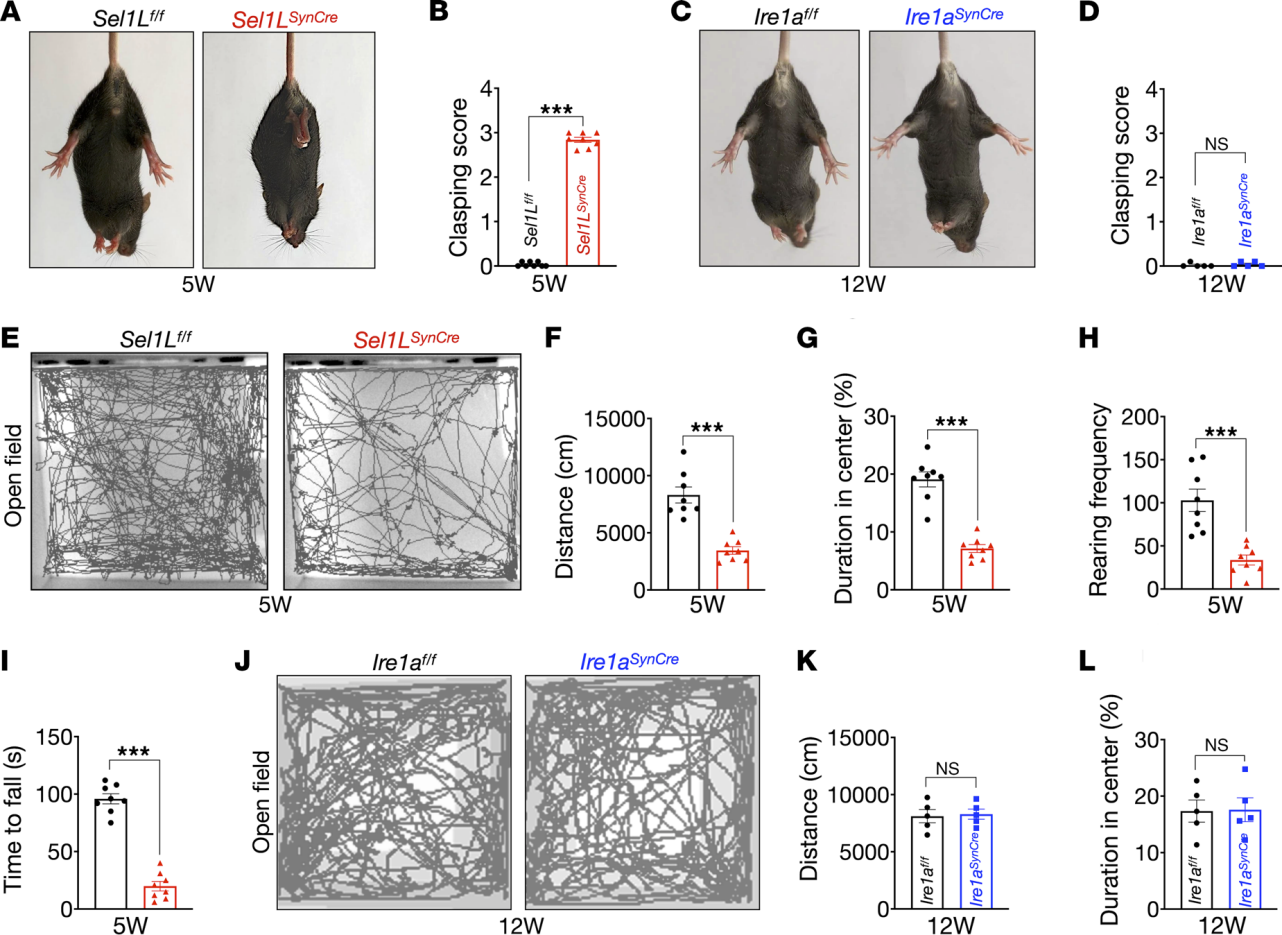
Impaired motor activity and behavior alterations of *Sel1L^SynCre^* mice but not *Ire1a*^SynCre^ mice. (**A**) Representative images of 5-week-old wild-type (*Sel1L*^fl/fl^) and neuronal *Sel1L*-deficient (*Sel1L^SynCre^*) male mice showing hind limb–clasping reflex. Clasping score quantitation is shown in **B** (5 weeks, *n* = 8 mice per group). (**C**) Representative images of 12-week-old wild-type (*Ire1a^fl/fl^*) and neuronal *Ire1a*-deficient (*Ire1a^SynCre^*) male mice. Clasping score quantitation is shown in **D** (12 weeks, *n* = 5 mice per group). (**E**) Open field test showing representative locomotor activity traces for *Sel1L*^fl/fl^ and *Sel1L^SynCre^* mice at 5 weeks of age. (**F**–**H**) Open field test quantification showing total distance (**F**), duration in the center (**G**), and rearing frequency (**H**). Animals were video recorded for 30 minutes and analyzed using EthoVision XT software (5 weeks, *n* = 8 for each genotype). (**I**) Motor performance on the accelerating rotarod test in *Sel1L*^fl/fl^ and *Sel1L^SynCre^* mice (5 weeks, *n* = 8 mice per group). (**J**–**L**) Open field test showing representative locomotor activity traces for *Ire1a^fl/fl^* and *Ire1a^SynCre^* mice at 12 weeks of age, with quantification of total distance (**K**) and duration in the center (**L**) (12 weeks, *n* = 5 mice per group). The data presented are shown as mean ± SEM and were analyzed using 2-tailed Student’s *t* test. Significance levels are indicated as follows: ***P < 0.001. NS, not significant.

*Sel1L^SynCre^* mice began to exhibit neurological abnormalities as early as 2 weeks of age (Supplemental Video 2). A prominent early phenotype was abnormal limb-clasping reflexes when suspended by the tail, in contrast with WT littermates, which showed normal limb extension (Figure 4, A and B). In comparison, *Ire1a^SynCre^* mice exhibited normal limb-clasping responses, even at 12 weeks of age (Figure 4, C and D). In the open field test, *Sel1L^SynCre^* mice demonstrated substantially reduced spontaneous locomotor activity (Figure 4E). Quantitative analysis over a 30-minute period revealed a 60% reduction in total distance traveled (Figure 4F) and a 63% decrease in time spent in the center of the arena, indicating increased anxiety-like behavior (Figure 4G). Additionally, these mice exhibited a 68% reduction in rearing frequency, an exploratory behavior in rodents (46), further supporting behavioral impairment (Figure 4H). Motor coordination was also compromised in *Sel1L^SynCre^* mice, as evidenced by an 80% reduction in latency to fall during rotarod testing (Figure 4I). In contrast, *Ire1a^SynCre^* mice displayed no significant deficits in locomotor activity or behavior (Figure 4, J–L). Collectively, these data indicate that SEL1L expression in neurons is essential for maintaining normal motor coordination and behavioral responses, including anxiety, in mice.

### Neuronal SEL1L deficiency does not induce significant cell death.

**Figure 5:**
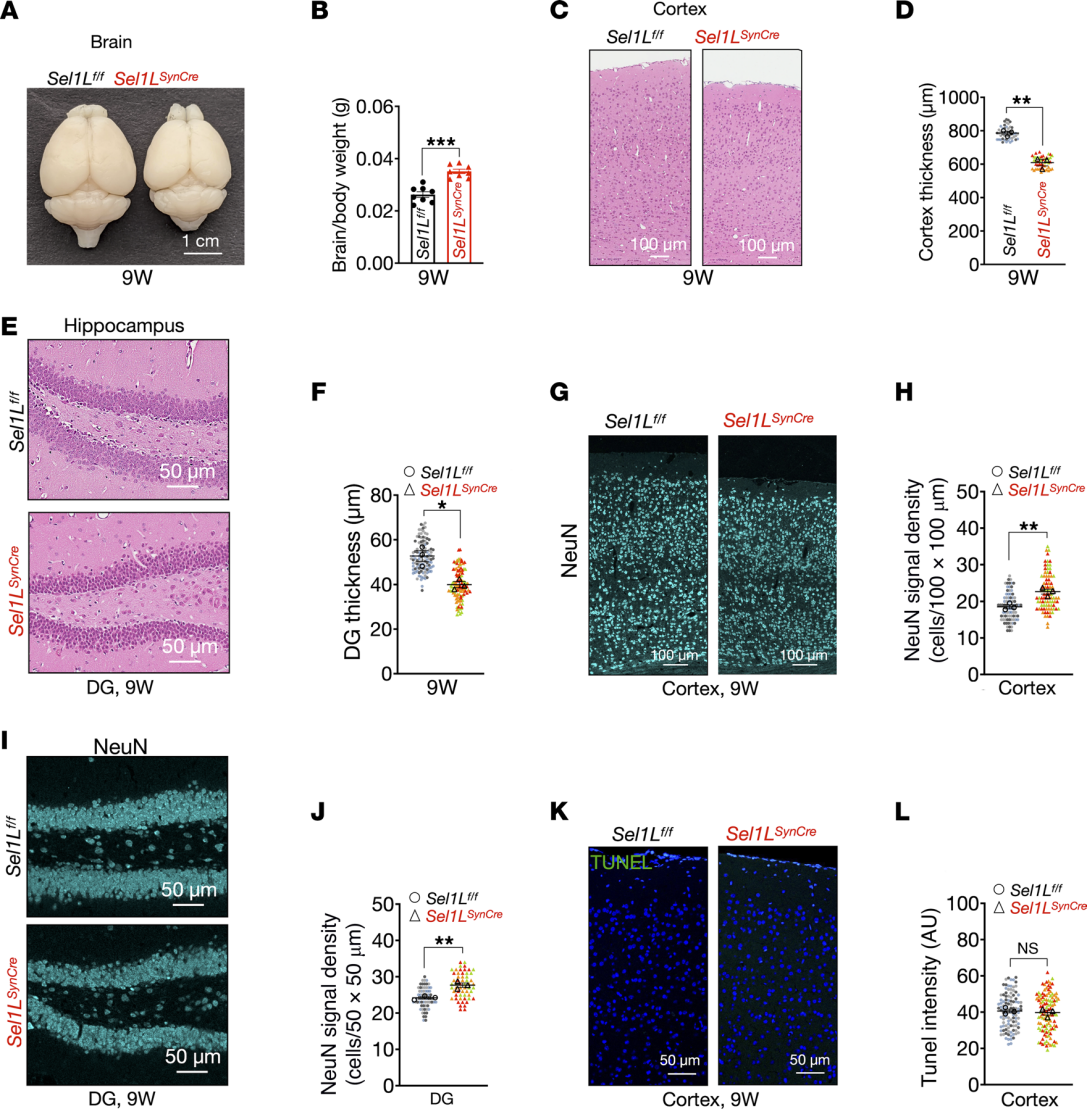
Neuronal SEL1L deficiency does not induce significant cell death. (**A**) Representative images of brains from *Sel1L*^fl/fl^ and *Sel1L^SynCre^* mice at 9 weeks of age. (**B**) Whole brain weight for both genotypes normalized by body weight (*n* = 8 mice per group). (**C**) Hematoxylin and eosin staining of the cerebral cortex from *Sel1L*^fl/fl^ and *Sel1L^SynCre^* mice at 9 weeks of age. Quantitation of cortical thickness is shown in **D** (50–60 measurements from 3 mice per group; data from each mouse are shown in different colors). (**E**) Hematoxylin and eosin staining of the DG region in the hippocampus at 9 weeks of age. Quantitation of the granular layer thickness in the DG is shown in **F** (50–60 measurements from 3 mice per group). (**G**) Representative confocal images of NeuN staining in cerebral cortex sections from *Sel1L*^fl/fl^ and *Sel1L^SynCre^* mice at 5 weeks of age. Quantitation of NeuN-positive cells’ (neurons) density in the cortex is shown in **H** (50–60 measurements from 3 mice per group). (**I**) Representative confocal images of NeuN staining in the DG region of the hippocampus at 5 weeks of age. Quantitation of NeuN-positive cells’ (neurons) density in the granular layer is shown in **J** (50–60 measurements from 3 mice per group). (**K**) Analysis of DNA fragmentation by TUNEL assay in cerebral cortex sections from *Sel1L*^fl/fl^ and *Sel1L^SynCre^* mice at 5 weeks of age. Quantification of the average signal within a 100 × 100 μm^2^ tissue area is shown in **L** (100–120 measurements from 3 mice per group). The data presented are shown as mean ± SEM and were analyzed using 2-tailed Student’s *t* test. Significance levels are indicated as follows: *P < 0.05; **P < 0.01; ***P < 0.001. NS, not significant.

To investigate the impact of SEL1L deficiency in neurons, we compared brain size between *Sel1L^SynCre^* mice and WT littermates. Although *Sel1L^SynCre^* mice exhibited a 22% reduction in absolute brain weight compared with WT littermates, brain weight normalized to body weight was comparable between groups (Figure 5, A and B). Histological analysis using H&E staining revealed an approximately 20% reduction in cortical thickness and DG size (granule cell layer) in *Sel1L^SynCre^* mice, consistent with reduced brain size (Figure 5, C–F). No significant changes were observed in the CA1 region, in line with the lack of SEL1L deletion there (Supplemental Figure 5, A and B). Despite these structural alterations, neuronal density assessed by immunofluorescence staining for the neuronal marker NeuN was slightly increased in both the cortex and DG of *Sel1L^SynCre^* mice (Figure 5, G–J). TUNEL assays showed no significant differences in DNA fragmentation between *Sel1L^SynCre^* and WT mice in either region (Figure 5, K and L, and Supplemental Figure 5, C and D). Furthermore, levels of cleaved caspase-3, a marker of apoptosis, were unchanged in the cortex and hippocampus (Supplemental Figure 5, E and F). Together, these findings indicate that neuronal SEL1L deficiency does not reduce neuronal density or induce apoptosis at 9 weeks of age.

### Mild activation of the UPR in Sel1L^SynCre^ mice.

**Figure 6:**
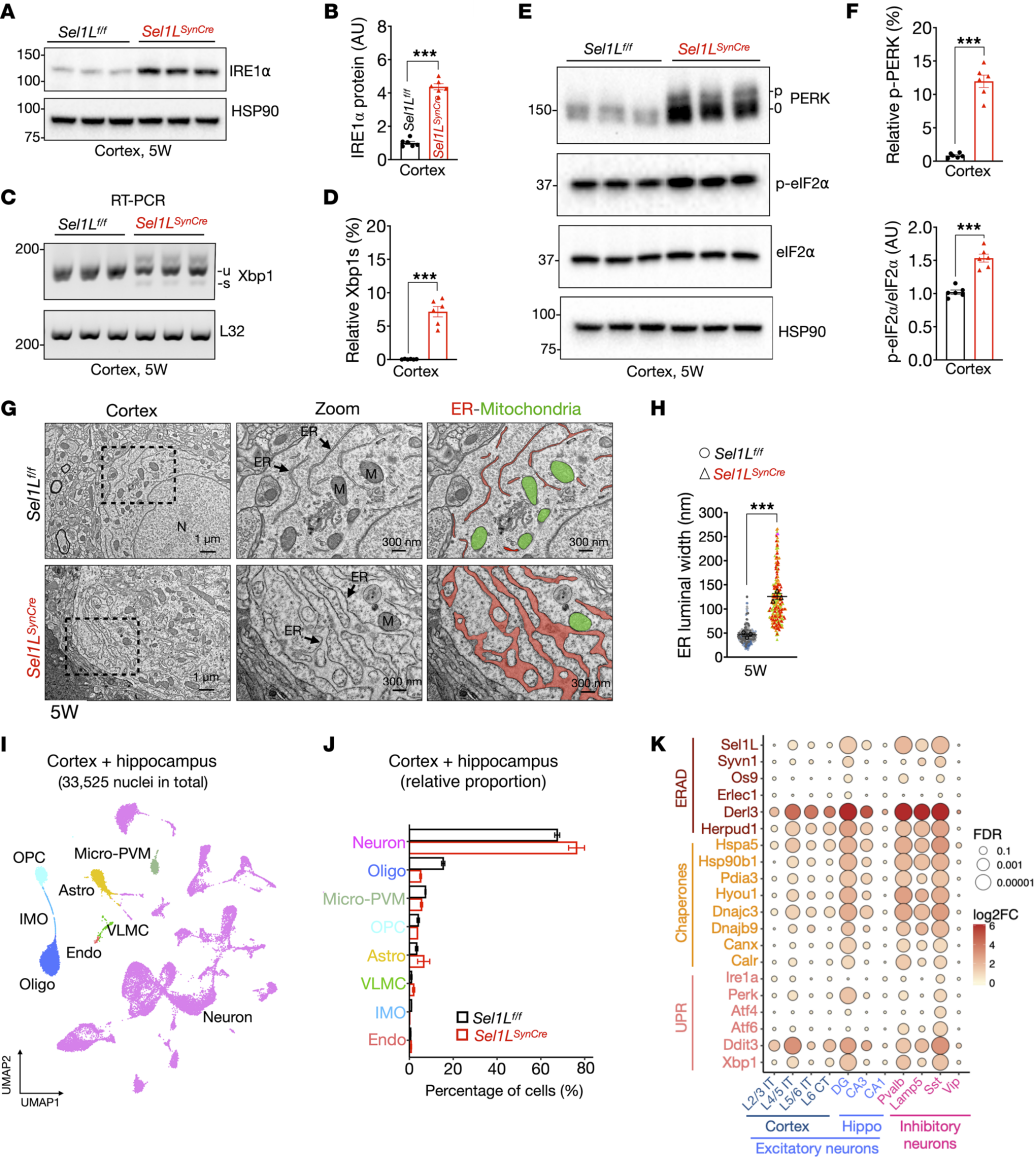
Partial UPR activation in *Sel1L*-deficient neurons. (**A**) Western blot analysis of IRE1α in cortical tissues of *Sel1L*^fl/fl^ and *Sel1L^SynCre^* mice at 5 weeks of age. (**B**) Quantitation is shown (*n* = 6 mice per group; molecular weight in kDa). (**C**) RT-PCR analysis of *Xbp1* mRNA splicing in cortical tissue from 5-week-old mice; u and s indicate the unspliced and spliced *Xbp1*, respectively. (**D**) Quantitation of spliced Xbp1 (Xbp1s) is shown (*n* = 6 mice per group). (**E**) Western blot analysis of PERK and phosphorylated eIF2α (p-eIF2α) in cortical tissue, with quantitation shown in **F** (*n* = 6 mice per group). (**G**) Representative TEM images from *Sel1L*^fl/fl^ and *Sel1L^SynCre^* mice at 5 weeks of age. ER (red) and mitochondria (M, green) are indicated. (**H**) Quantitation of ER luminal width is shown (200–250 measurements from 3 mice per group). (**I**) Uniform manifold approximation and projection (UMAP) visualization of cell types identified by unsupervised clustering of snRNA-seq data from combined cortex and hippocampus. Each dot represents 1 nucleus. (**J**) Relative abundance of major cell types between genotypes. Cell types include neurons, oligodendrocytes (Oligo), microglia and perivascular cells (Micro-PVM), oligodendrocyte precursor cells (OPC), astrocytes (Astro), vascular and leptomeningeal cells (VLMC), inflammatory mononuclear cells (IMO), and endothelial cells (Endo). (**K**) Differential expression of ER quality control–related genes (ERAD, chaperones, and the UPR pathways) based on snRNA-seq data, shown as log_2_ fold change. Excitatory and inhibitory neuronal subtypes were defined using established markers. Data in **B**, **D**, **F**, and **H** are mean ± SEM and analyzed by 2-tailed Student’s *t* test; data in **K** were analyzed by 2-way ANOVA with FDR correction. ***P < 0.001.

To assess ER homeostasis in *Sel1L^SynCre^* mice, we evaluated the expression and activity of key UPR sensors and their downstream effectors, as previously described (47, 48). Western blot analysis revealed a marked increase in IRE1α protein levels in *Sel1L^SynCre^* cortical lysates compared with WT controls (Figure 6, A and B), consistent with prior findings that IRE1α is a substrate of SEL1L-HRD1 ERAD (49). IRE1α-mediated splicing of *Xbp1* mRNA was elevated in *Sel1L^SynCre^* mice, increasing from ~1% in WT mice to ~7% in Sel1L-deficient mice (Figure 6, C and D), indicative of modest activation of the IRE1α arm of the UPR. Activation of the PERK pathway was also modest, as evidenced by a modest increase in PERK and eIF2α phosphorylation (Figure 6, E and F). Transmission electron microscopy (TEM) revealed a ~3-fold increase in ER lumen width in neurons from *Sel1L^SynCre^* mice, indicative of ER dilation (Figure 6, G and H, and Supplemental Figure 6). These findings indicate mild UPR activation, accompanied by ultrastructural changes in the ER.

### snRNA-seq reveals cellular adaptation in Sel1L^SynCre^ neurons.

To further investigate the impact of Sel1L deletion at single-cell resolution, we performed snRNA-seq on cortical and hippocampal tissues from *Sel1L^SynCre^* and WT mice. A total of 33,525 nuclei were analyzed and classified into major brain cell types, including neurons, oligodendrocytes, microglia/perivascular macrophages, oligodendrocyte precursor cells (OPCs), and astrocytes, based on canonical marker genes (Figure 6I and Supplemental Figure 7, A and B) (40). While the overall distribution of cell types was similar between genotypes, *Sel1L^SynCre^* mice exhibited a modest increase in neuronal representation and a reduction in oligodendrocyte proportions, with astrocyte and microglial populations remaining unchanged (Figure 6J).

We next subclustered neuronal nuclei from cortex and hippocampus and annotated their regional and cellular identities using well-established transcriptomic markers (Supplemental Figure 7, C and D) (40). Among inhibitory neurons, GABAergic subtypes expressing *Pvalb*, *Sst*, and *Lamp5* exhibited the most pronounced transcriptional alterations in *Sel1L^SynCre^* mice (Supplemental Figure 8, A and B). Within excitatory populations, deep-layer cortical projection neurons (L4/5 IT CTX) and DG granule cells were among the most affected subtypes (Supplemental Figure 8, A and B). Despite these transcriptional changes in multiple excitatory and inhibitory neuronal clusters, we did not observe marked differences in the relative abundance of these neuronal subtypes between *Sel1L^fl/fl^* and *Sel1L^SynCre^* mice (Supplemental Figure 8C). Additionally, we assessed expression of genes involved in ER protein quality control in clusters containing excitatory and inhibitory neurons (Figure 6K). Several ERAD genes, including *Derl3* (Derlin-3) and *Herpud1*, were upregulated, along with chaperones such as *Hspa5* (BiP), *Hsp90b1* (GRP94), and *Hyou1* (GRP170), particularly in DG granule cells and *Pvalb^+^* and *Sst^+^* inhibitory neurons, where the response to ERAD deficiency appeared more pronounced (Figure 6K). Additional ER chaperones, including *Canx* (calnexin), *Calr* (calreticulin), and *Pdia3* (ERp57), were upregulated in the same neuronal populations (Figure 6K). Moreover, select UPR components, such as *Eif2ak3* (PERK), *Xbp1*, and *Ddit3* (CHOP), were elevated, whereas others, including *Ern1* (*Ire1a*), activating transcription factor 6 (*Atf6*), and *Atf4*, were unchanged (Figure 6K). Together, these data uncover a cell type–specific transcriptional response to ERAD deficiency in *Sel1L^SynCre^* mice, marked by upregulation of ER protein quality control pathways in distinct excitatory and inhibitory neuronal populations.

### Neuronal Sel1L deficiency activates one-carbon metabolism.

**Figure 7:**
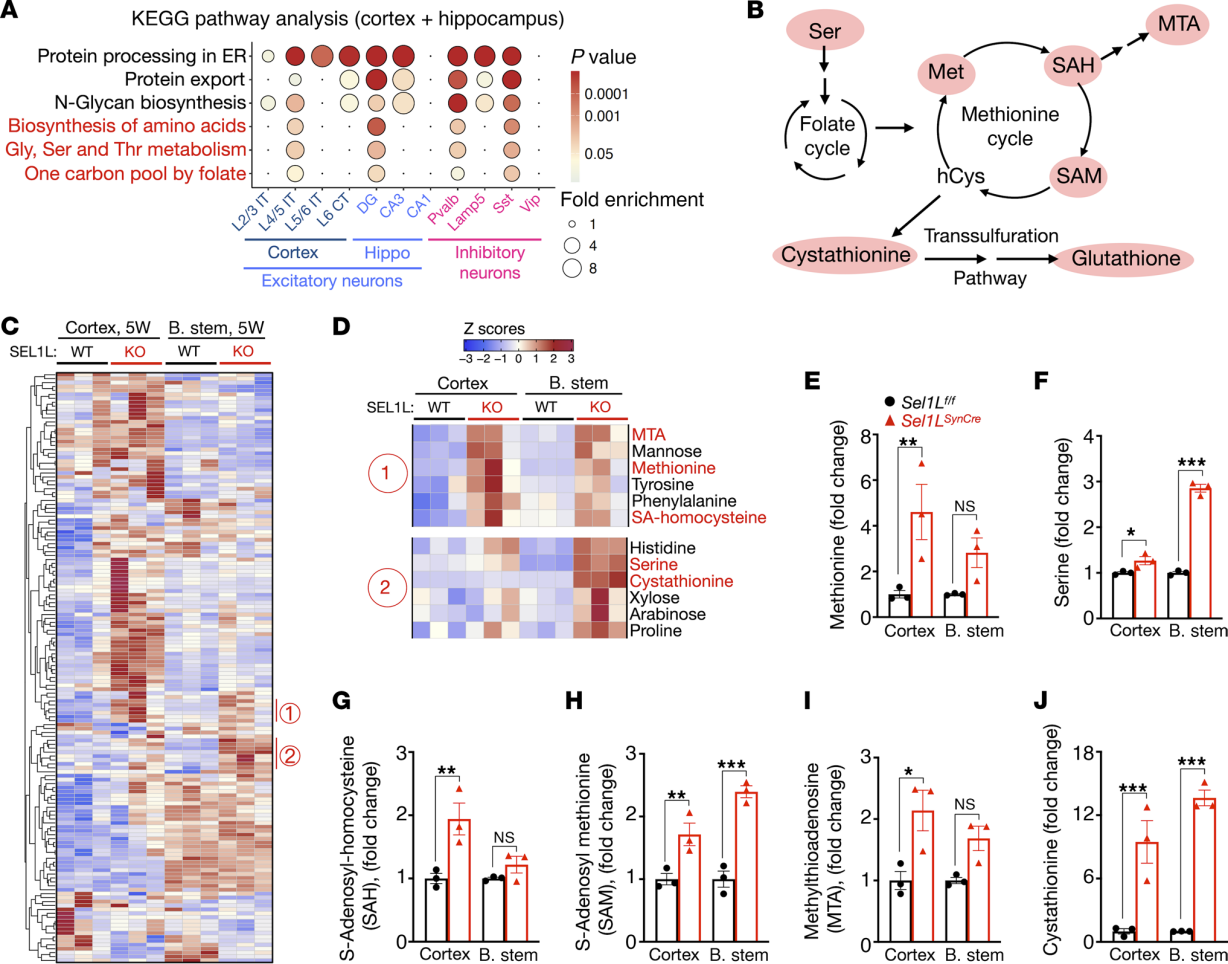
Upregulation of one-carbon metabolism pathways in the brain of *Sel1L^SynCre^* mice. (**A**) KEGG pathway enrichment analysis of differentially expressed genes in *Sel1L^SynCre^* mice, separated by neuronal subtypes isolated from the cortex and hippocampus at 5 weeks of age. (**B**) Diagram illustrating one-carbon metabolism, including folate, methionine, and transsulfuration pathways. Key metabolites analyzed include methionine (Met), serine, *S*-adenosyl-homocysteine (SAH), *S*-adenosyl methionine (SAM), methylthioadenosine (MAT), cystathionine, and glutathione. (**C**–**J**) Metabolomics analysis of cortex and brain stem tissues isolated from *Sel1L*^fl/fl^ (WT) and *Sel1L^SynCre^* (KO) mice at 5 weeks of age. Panel **D** highlights selected regions showing changes in metabolites related to one-carbon metabolism. (**E**–**J**) Fold-change plots showing individual metabolites from the cortex and brain stem of *Sel1L*^fl/fl^ and *Sel1L^SynCre^* mice (*n* = 3 animals per brain region). The data presented are shown as mean ± SEM and were analyzed using a 2-way ANOVA with FDR correction. Significance levels are indicated as follows: **P < 0.01; ***P < 0.001. NS, not significant.

To identify pathways dysregulated by neuronal loss of *Sel1L*, we performed Kyoto Encyclopedia of Genes and Genomes (KEGG) pathway enrichment analysis using our snRNA-seq dataset from cortical and hippocampal regions. In addition to expected upregulation of pathways related to protein synthesis and ER quality control, we observed significant enrichment of one-carbon metabolism and amino acid biosynthesis pathways, including biosynthesis of amino acids; glycine, serine, and threonine metabolism; and one carbon pool by folate (highlighted in red, Figure 7A). One-carbon metabolism includes the folate cycle, methionine cycle, and transsulfuration pathway (Figure 7B), which collectively support methylation reactions, nucleotide synthesis, and redox homeostasis (36).

To validate these findings, we performed metabolomic profiling in 2 anatomically distinct brain regions: the cortex and the brain stem (pons and medulla). Deletion of SEL1L in the brain stem was confirmed by Western blot (Supplemental Figure 9, A and B). Notably, heatmap analysis revealed a consistent increase in metabolites associated with one-carbon metabolism in both regions of *Sel1L^SynCre^* mice compared with WT controls (Figure 7, C and D). Specifically, methionine, serine, *S*-adenosylhomocysteine (SAH), *S*-adenosylmethionine (SAM), methylthioadenosine (MTA), and cystathionine were significantly increased (Figure 7, E–J). In contrast, levels of both reduced and oxidized glutathione remained unchanged in the cortex and brain stem (Supplemental Figure 9, C and D). These metabolite changes suggest alterations in methylation capacity, redox balance, and epigenetic regulation, key features associated with neurometabolic and neurodevelopmental disorders (50). Together, these results indicate that neuronal *Sel1L* deficiency induces broad activation of one-carbon metabolism.

### Activation of ATF4 and one-carbon metabolic genes in Sel1L^SynCre^ neurons.

**Figure 8:**
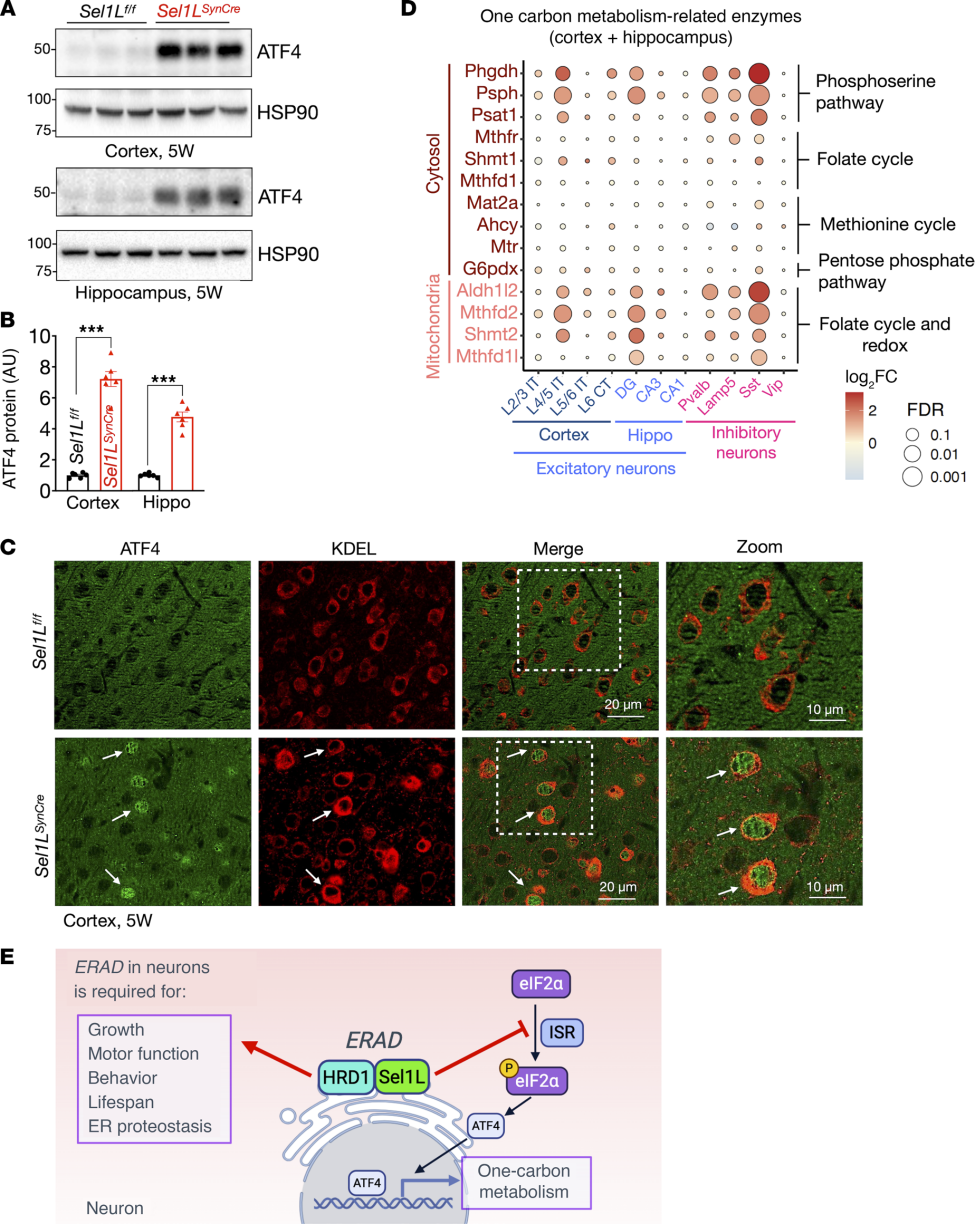
Activation of ATF4 and one-carbon metabolism enzymes in the brain of *Sel1L^SynCre^* mice. (**A**) Western blot analysis of ATF4 in cortical and hippocampal tissues from *Sel1L*^fl/fl^ and *Sel1L^SynCre^* mice at 5 weeks of age, with quantitation shown in **B** (*n* = 6 mice per group). (**C**) Representative confocal images of ATF4 (green) and KDEL (red) in the cortex of 5-week-old *Sel1L^SynCre^* mice. (**D**) Relative mRNA abundance of one-carbon metabolism genes based on single-nucleus RNA sequencing of cortical and hippocampal tissue from *Sel1L*^fl/fl^ and *Sel1L^SynCre^* mice at 5 weeks of age. (**E**) Proposed model illustrating the role of ERAD in neurons. Proper ERAD function is essential for maintaining growth, motor function, behavior, and lifespan in mice by preserving ER proteostasis under basal conditions and preventing activation of the ISR. Loss of ERAD leads to phosphorylation of eIF2α, increased ATF4 levels, and activation of one-carbon metabolism pathways. Positive and inhibitory regulatory effects are indicated by red arrow and blunt-ended line, respectively. Data in **B** are presented as mean ± SEM and differences between *Sel1L^fl/fl^* and *Sel1L^SynCre^* groups were analyzed using 2-tailed Student’s *t* test. The data presented (**C**) were analyzed using a 2-way ANOVA with FDR correction. Significance levels are indicated as follows: ***P < 0.001.

The ISR, particularly the eIF2α/ATF4 axis, plays a central role in adapting to cellular stress by modulating amino acid metabolism and antioxidant responses (29, 51). Consistent with the ISR activation, we observed an approximately 40% increase in eIF2α phosphorylation (Figure 6, E and F), along with 7-fold and 5-fold elevation in ATF4 protein levels in the cortex and hippocampus of *Sel1L^SynCre^* mice, respectively (Figure 8, A and B). Confocal analysis of ATF4 and KDEL in cortical and hippocampal tissues of 5-week-old mice further supported ISR activation, showing increased nuclear ATF4 abundance in *Sel1L*-deficient neurons (Figure 8C and Supplemental Figure 10).

As ATF4 is also a known transcription regulator of enzymes involved in one-carbon metabolism (52, 53), we next analyzed the expression of one-carbon metabolism genes in the snRNA-seq dataset (Figure 8D). Mitochondrial genes including *Mthfd2* (methylenetetrahydrofolate dehydrogenase 2) and *Shmt2* (serine hydroxymethyltransferase 2) were significantly upregulated, along with moderate increases in *Adh1l2* (alcohol dehydrogenase 1-like protein 2) and *Mthfd1l* (methylenetetrahydrofolate dehydrogenase 1-like) (Figure 8D). Genes in the phosphoserine pathway, *Phgdh* (phosphoglycerate dehydrogenase), *Psph* (phosphoserine phosphatase), and *Psat1* (phosphoserine aminotransferase 1), were also elevated, consistent with increased serine biosynthesis from glucose (54). In contrast, expression of genes involved in cytosolic folate and methionine metabolism — including *Mthfr* (methylenetetrahydrofolate reductase), *Shmt1* (serine hydroxymethyltransferase 1), *Mthfd1* (methylenetetrahydrofolate dehydrogenase 1), *Mat2a* (methionine adenosyltransferase 2A), *Ahcy* (adenosylhomocysteinase), and *Mtr* (methionine synthase) — as well as *G6pdx* (glucose-6-phosphate dehydrogenase X-linked), a key enzyme in the pentose phosphate pathway (55), remained unchanged (Figure 8D). These data support a model in which neuronal *Sel1l* deficiency activates the ATF4 pathway and selectively enhances mitochondrial folate cycle and serine biosynthesis of the one-carbon metabolism pathway (Figure 8E).

## Discussion

Our findings establish that the SEL1L-HRD1 ERAD complex is essential for central nervous system function, contributing to normal growth, behavior, motor control, and survival in mice. In *Sel1L^SynCre^* mice, we observed neurological impairments and early death, accompanied by a marked activation of the one-carbon metabolism pathway — revealing an unexpected link between ERAD deficiency, neuronal dysfunction, and metabolic reprogramming. These findings, together with recent reports of SEL1L-HRD1 disease variants in patients with ENDI (26, 27) and studies involving conditional deletion of *Sel1l* in adult neurons (56), underscore the essential role of this ER quality control pathway in sustaining neuronal homeostasis. Moreover, we demonstrate that SEL1L deficiency leads to only mild activation of the UPR and that neuron-specific deletion of *Ire1a* using the same Cre driver fails to recapitulate the motor and behavioral deficits seen in *Sel1L^SynCre^* mice. These results suggest that UPR activation alone is insufficient to explain the observed phenotypes and point to additional substrate-dependent mechanisms underlying neuronal vulnerability.

Motor impairment is a consistent phenotype in ERAD-deficient models (56, 57), suggesting that motor neurons and associated neural circuits are particularly dependent on intact ERAD function. While previous work demonstrated that PC-specific deletion of *Sel1L* results in progressive cerebellar ataxia and neurodegeneration (35), we did not detect a marked reduction of SEL1L in cerebellar PCs in *Sel1L^SynCre^* mice, indicating relative sparing of the cerebellum in this model. Previous studies have shown that the Synapsin-Cre promotor is also active in the spinal cord (41). Our preliminary analyses of the spinal cord in the *Sel1L^SynCre^* model indicate loss of ERAD function in the ventral, intermediate, and posterior regions, with preservation of motor neurons numbers (data not shown), suggesting a potential contribution of somatic motor neurons to the motor phenotype. Generation of a motor neuron–specific ERAD-deficient mouse model will be required to assess the direct contribution of motor neurons to the motor phenotype and to determine whether metabolic alterations in these neurons correlate with the observed motor dysfunction.

In addition, snRNA-seq analysis revealed a selective reduction in mature oligodendrocytes in *Sel1L^SynCre^* mice, whereas OPCs were largely preserved. Given that neuronal activity promotes OPC differentiation and myelination (58, 59), these findings raise the possibility that neuronal dysfunction may impair oligodendrocyte maturation. In our snRNA-seq data, we identified several differentially expressed genes in OPCs and oligodendrocytes; however, we did not detect significant differences in myelin basic protein levels in total spinal cord lysates (data not shown), suggesting that myelination is preserved in this tissue. Future studies focusing on neuron/glia signaling, myelin ultrastructure, and circuit-level connectivity will be essential to dissect the multifactorial basis of motor impairment in the context of neuronal ERAD deficiency.

By integrating 2 complementary unbiased omics approaches, snRNA-seq and metabolomics, we identified a prominent molecular hallmark of *Sel1L^SynCre^* mice: robust activation of one-carbon metabolism. This pathway is critical for nucleotide biosynthesis, amino acid metabolism, redox homeostasis, and epigenetic regulation (36). Notably, elevated levels of SAM and SAH suggest a disruption in methylation balance (60). As SAM is the universal methyl donor and SAH a potent methyltransferase inhibitor, an increased SAH/SAM ratio is often associated with global DNA hypomethylation and broad transcriptional dysregulation (61). The accumulation of upstream metabolites, including serine, methionine, and cystathionine, further indicates perturbations in redox and methylation dynamics (62), which may contribute to the neurological deficits observed in *Sel1L^SynCre^* mice. Although activation of the PERK/ATF4 pathway may contribute to aspects of this metabolic shift, we cannot exclude contributions from other ISR arms, such as GCN2, which is activated by amino acid deprivation, or HRI, which senses mitochondrial dysfunction (63), as well as the potential role of oxidative stress (64). Whether these metabolic alterations represent compensatory responses to chronic proteostatic stress or actively drive disease pathogenesis remains unresolved. Importantly, we were unable to replicate this one-carbon metabolic signature in ERAD-deficient mouse neuroblastoma cell lines, despite robust ATF4 accumulation (data not shown). This discrepancy suggests that ERAD-dependent metabolic reprogramming may require additional cell type–specific or in vivo contextual factors beyond acute ISR activation. Together, our findings reveal a critical role for the SEL1L-HRD1 ERAD complex in regulating neuronal metabolic homeostasis and identify one-carbon metabolism as a potential therapeutic target. This model provides a powerful platform for dissecting the molecular and metabolic basis of ERAD-associated neurodevelopmental disorders and for evaluating targeted metabolic or genetic interventions.

## Methods

### Sex as a biological variable.

Our study examined male and female animals, and similar findings are reported for both sexes.

### Mice.

Neuron-specific *Sel1L*-deficient mice (*Sel1L1^SynCre^*) were generated by breeding the Synapsin I-Cre mice on the C57BL/6J background (Jackson Laboratory 003966) with the *Sel1L^fl/fl^* mice on the C57BL/6J background (1). Age- and sex-matched littermates were housed in a temperature-controlled room on a 12-hour light/dark cycle.

### Genotyping.

*Sel1L^SynCre^* and *Ire1a^SynCre^* mice were routinely genotyped using PCR of genomic DNA samples obtained from ears with the following primer pairs: *Sel1L*^fl/fl^: F: 5′-CTGACTGAGGAAGGGTCTC-3′, R: 5′-GCTAAAAACATTACAAAGGGGCA-3′; *Ire1a^fl/fl^*: F: 5′-CCGAGCCATGAGAAACAAGG-3′, R: 5′-CCCTGCCAGGATGGTCATGG-3′; Cre recombinase: F: 5′-ACCTGAAGATGTTCGCGATTATCT-3′, R: 5′-ACCGTCAGTACGTGAGATATCTT-3′.

### Behavioral studies.

All behavior procedures were performed by investigators without knowledge of the genotypes. For hind limb–clasping assessment, mice were lifted by the tail and held over a cage for 1 minute to assess abnormal hind limb clasping, and scoring was performed as previously described (2). For the open field test, mice were placed in the center of the arena, and video recording began 3 seconds after the mouse was detected. Behavior was recorded for 30 minutes. At the end of the session, mice were returned to their home cage, and the apparatus was cleaned between trials. Total distance traveled, time spent in different zones, rearing, and movement were quantified using EthoVision XT (Noldus). For the rotarod test, an accelerating rotarod (LE8500, Panlab SL) was used. Mice were trained in 1 session per day over 4 consecutive days. The rotarod started at 4 rpm and accelerated to 40 rpm at a rate of 0.2 rpm/s. The latency to fall was recorded automatically, and performance on the fifth day was analyzed using a 3-minute cutoff.

### Western blot and antibodies.

Tissues were harvested and snap-frozen in liquid nitrogen. Proteins were extracted by sonication in 1% Triton X-100 buffer (50 mM Tris-HCl at pH 7.5, 150 mM NaCl, 1% Triton X-100, 1 mM EDTA) supplemented with protease inhibitor (Sigma), 1 mM DTT (Sigma), and phosphatase inhibitor cocktail (Sigma). Lysates were incubated on ice for 20 minutes and centrifuged at 16,000*g* for 10 minutes. Supernatants were collected, and protein concentrations were determined using the Bio-Rad Protein Assay Dye. A total of 20–30 μg of protein was denatured at 95°C for 5 minutes in 1× SDS sample buffer (50 mM Tris-HCl pH 6.8, 2% sodium dodecyl sulfate, 0.01% bromophenol blue, 10% glycerol, and 0.3 M β-mercaptoethanol). Proteins were separated by SDS-PAGE and transferred onto PVDF membranes (Thermo Fisher Scientific). Membranes were blocked in 2% BSA in Tris-buffered saline with 0.1% Tween 20 (TBST) and incubated overnight at 4°C with the following primary antibodies: anti-HSP90 (Santa Cruz Biotechnology, sc-7947, 1:5,000), anti-SEL1L (Abcam, ab78298, 1:1,000), anti-HRD1 (Proteintech, 13473-1, 1:2,000), anti-OS9 (Abcam, ab109510, 1:5,000), anti-IRE1α (Cell Signaling Technology, 3294, 1:2,000), anti-PERK (Cell Signaling Technology, 3192, 1:1,000), anti–p-eIF2α (Cell Signaling Technology, 9721, 1:1,000), anti-eIF2α (Cell Signaling Technology, 9722, 1:1,000), and anti-ATF4 (Cell Signaling Technology, 11815, 1:1,000). After washing with TBST, membranes were incubated with HRP-conjugated secondary antibodies (Bio-Rad,1706515, 1:5,000) for 1 hour at room temperature. Signals were developed using an ECL chemiluminescence detection system (Bio-Rad), and band intensities were quantified using ImageJ software (NIH).

### RNA preparation and RT-PCR.

Total RNA was extracted from tissues using TRI Reagent and BCP Phase Separation Reagent according to the manufacturer’s protocol (Molecular Research Center, TR118). RT-PCR for Xbp1 mRNA splicing was performed as previously described (3). The ratio of Xbp1s to total Xbp1 (Xbp1u + Xbp1s) was quantified by ImageJ software. RT-PCR primer sequences were mXbp1 F: ACGAGGTTCCAGAGGTGGAG, R: AAGAGGCAACAGTGTCAGAG; mL32 F: GAGCAACAAGAAAACCAAGCA, R: TGCACACAAGCCATCTACTCA.

### Histology.

Anesthetized mice were perfused with 20 mL of 0.9% NaCl followed by 40 mL of 4% paraformaldehyde in 0.1 M PBS (pH 7.4) for fixation. Brains were dissected and postfixed overnight in 4% paraformaldehyde in PBS at 4°C. For H&E staining, tissues were dehydrated, embedded in paraffin, and processed at the Rogel Cancer Center Tissue and Molecular Pathology Core at the University of Michigan. Cortical and hippocampal thickness was quantified on H&E-stained coronal sections using Aperio ImageScope software.

### Immunofluorescence staining.

Paraffin-embedded brain sections were deparaffinized in xylene and rehydrated through a graded ethanol series (100%, 90%, 70%), followed by rinsing in distilled water. Antigen retrieval was performed by boiling the slides in a microwave using a citric acid–based antigen unmasking solution (Vector Laboratories, H-3300). Sections were then incubated in blocking solution (5% donkey serum, 0.3% Triton X-100 in PBS) for 1 hour at room temperature, followed by overnight incubation at 4°C in a humidified chamber with the following primary antibodies: anti-NeuN (EMD Millipore, ABN90, 1:500), anti-GFAP (Cell Signaling Technology, 3670, 1:100), anti-KDEL (Novus Biologicals, 97469, 1:200), anti-SEL1L (Abcam, ab78298, 1:100), and anti-ATF4 (Cell Signaling Technology, 11815, 1:100). The next day, after 3 washes with 0.03% Triton X-100 in PBS, sections were incubated with the corresponding Alexa Fluor–conjugated secondary antibodies (Jackson ImmunoResearch, 711565152, 1:500) for 1 hour at room temperature. Slides were then mounted with VECTASHIELD mounting medium containing DAPI (Vector Laboratories, H-1500). Images were acquired using a Nikon A1 confocal microscope at the University of Michigan Microscopy and Image Analysis Core. Images were analyzed using ImageJ software.

### TUNEL assay.

Paraffin-embedded brain sections were processed for TUNEL staining using the In-Situ Cell Death Detection Kit (Roche, 11684795910) according to the manufacturer’s instructions. Images were acquired using a Nikon A1 confocal microscope at the University of Michigan Microscopy and Image Analysis Core. Quantification was performed using ImageJ software.

### TEM.

Mice were anesthetized and perfused with 3% glutaraldehyde and 3% formaldehyde in 0.1 M cacodylate buffer (Electron Microscopy Sciences, 16220, 15710, 11653). Cortical brain regions were dissected and cut into small pieces, then postfixed overnight at 4°C in 3% glutaraldehyde, 3% formaldehyde in 0.1 M Sorenson’s buffer (Electron Microscopy Sciences, 11682). Tissue samples were then processed, embedded, and sectioned at the University of Michigan Microscopy Core. Sections were stained with uranyl acetate and lead citrate, and high-resolution images were acquired using a JEOL 1400-plus transmission electron microscope.

### Tissue preparation, cDNA amplification, and library construction for 10x Genomics snRNA-seq.

Mice were euthanized using isoflurane and decapitated. Brains were removed from the skull and sectioned into 1 mm sagittal slices using a brain matrix. The cortex and hippocampus from 2 *Sel1L^fl/fl^* and 2 *Sel1L^SynCre^* 5-week-old mice were dissected and flash-frozen in liquid nitrogen. On the day of the experiment, frozen tissues were homogenized in lysis buffer (EZ Prep Nuclei Kit, Sigma-Aldrich) supplemented with Protector RNase Inhibitor (Sigma-Aldrich) and filtered through a 30 μm MACS strainer (Miltenyi Biotec). Filtered samples were centrifuged at 500*g* for 5 minutes at 4°C, and the nuclear pellet was resuspended in wash buffer (10 mM Tris buffer at pH 8.0, 5 mM KCl, 12.5 mM MgCl_2_, 1% BSA with RNase inhibitor). Nuclei were filtered again and centrifuged under the same conditions. The resulting nuclei were resuspended in wash buffer containing propidium iodide (Sigma-Aldrich), and stained nuclei were sorted using a MoFlo Astrios Cell Sorter. Sorted nuclei were centrifuged at 100*g* for 5 minutes at 4°C and resuspended in wash buffer to a final concentration of 750–1,200 nuclei/μL. Reverse transcription mix was added to target approximately 10,000 nuclei, which were then loaded onto a 10x Genomics Chromium Controller chip. The Chromium Single Cell 3′ Library and Gel Bead Kit v3, Chromium Chip B Single Cell kit, and Chromium i7 Multiplex Kit were used for RT, cDNA amplification, and library preparation according to the manufacturer’s instructions. Libraries were sequenced on an Illumina NovaSeq 6000 platform with 150 nt paired-end reads.

### snRNA-seq data analysis.

Raw sequencing reads were processed through demultiplex, mapping, and analysis by the pipeline in 10x Genomics Cell Ranger, and the output data were further analyzed including integration using the Seurat package (version 4.3.0) in the R environment (version 4.2.3). After removing doublets and cells with low quality (high mitochondrial content or low sequencing depth), 33,636 cells that expressed more than 600 genes and 23,886 genes with transcripts detected in more than 3 cells were used for further analysis. The average unique molecular identifier count for each cell was 9,216. Unsupervised clustering was applied at a resolution of 0.2 using the top 17 dimensions of PCA using the default pipeline in the Seurat package. Cell cluster identification was based on the prior knowledge of marker genes (65). Differential gene expression analysis was conducted for each cluster between genotypes with the pseudo-bulking method using the edgeR package (version 4.4.0). The UMAP plots, feature plots, and heatmaps were generated in R.

### Metabolomics.

Mice were euthanized using isoflurane and decapitated. Brains were removed from the skull and sectioned into 1 millimeter–thick sagittal slices using a brain matrix. The cortex and hippocampus were dissected, flash-frozen in liquid nitrogen, and stored at –80°C. On the day of the experiment, frozen tissue samples were homogenized in cold (–80°C) 80% methanol. Soluble metabolites were separated from the insoluble fraction by centrifugation (16,000*g* for 10 min at 4ºC) and dried using a SpeedVac, with volumes normalized to tissue weights. Dried metabolites were reconstituted in a 1:1 methanol/water solution for LC-MS analysis. Metabolite extracts were analyzed using an Agilent Technologies Triple Quadrupole 6470 LC/MS system, equipped with a 1290 Infinity II LC Flexible Pump (Quaternary Pump), the 1290 Infinity II Multisampler, a 1290 Infinity II Multicolumn Thermostat with 6-port valve, and a 6470 triple quadrupole mass spectrometer. Agilent MassHunter Workstation Software (LC/MS Data Acquisition for 6400 Series Triple Quadrupole MS with Version B.08.02) was used for compound optimization, calibration, and data acquisition. Extracted ion chromatograms and mass spectra were manually inspected to ensure sample quality and consistent peak integration. Pathway analysis of significantly altered metabolites was performed using MetaboAnalyst (https://www.metaboanalyst.ca). Unsupervised hierarchically clustered heatmaps were generated by using R software.

### Statistics.

Statistical analyses were performed in Prism version 8.0 (GraphPad Software). Unless indicated otherwise, values are presented as mean ± SEM. All experiments have been repeated at least 2 to 3 times and/or performed with multiple independent biological samples from which representative data are shown. All datasets passed normality and equal variance tests. Statistical differences between groups were assessed using an unpaired 2-tailed Student’s *t* test for comparisons between 2 groups or a 2-way ANOVA followed by FDR correction for multiple comparisons. A *P* value of less than 0.05 was considered statistically significant.

### Study approval.

All animal procedures were approved by the Institutional Animal Care and Use Committee of the University of Michigan Medical School (PRO00010658) and the University of Virginia (PRO0004459), in accordance with the NIH Guide for the Care and Use of Laboratory Animals (National Academies Press, 2011).

### Data availability.

All materials and reagents used in this study are either commercially available or available upon request from the corresponding authors. Raw sequencing data for the snRNA-seq experiment have been deposited in NCBI GEO database under accession number GSE299894. All data are included within the figures, and the quantitative data presented in the figures are provided in the Supporting Data Values file.

## Author contributions

MT designed and performed most experiments; JH established the original *Sel1L^SynCre^* colony; HW, BP, XC, LLL, AJT, and AG contributed to both in vitro and in vivo experiments; YL and ACR performed the snRNA-seq analysis; AJS assisted with the metabolomics analysis; MGM, ZZ, CAL, and DRW provided insightful discussion; and LQ directed the study. LQ and MT wrote the manuscript; all authors commented on and approved the manuscript.

## Conflict of interest

The authors have declared that no conflict of interest exists.

## Funding support

This work is the result of NIH funding and is subject to the NIH Public Access Policy. Through acceptance of this federal funding, the NIH has been given a right to make the work publicly available in PubMed Central.

- NIH grant R01NS138119 (to LQ).
- NIH grant R01AG089640 (to LQ and ZZ).
- NIH grant RF1NS122060 (to ZZ).
- Alzheimer’s Association 24AARG-D-NTF-1187603 (to LQ).
- NCI F32CA260735 (to AJS).
- P30 grants DK020572, DK089503, and 1U2CDK135066 (to University of Michigan Animal Phenotyping Core).
- NIH NIDDK grant P30DK020572 (to University of Michigan Microscopy and Image Analysis Core).

## Supplementary Material

Supplemental data

Unedited blot and gel images

Supplemental video 1

Supplemental video 2

Supporting data values

## Version 1 02/19/2026

In-Press Preview

## Version 2 04/15/2026

Electronic publication
