# Supplementary material for: Neuronal SEL1L-HRD1 ER-associated degradation is essential for motor function and survival in mice
Source: J Clin Invest. 2026 Feb 19;136(8):e196819. doi: 10.1172/JCI196819 (PMC13078874; doi:10.1172/JCI196819)

Figure 6A and E

A

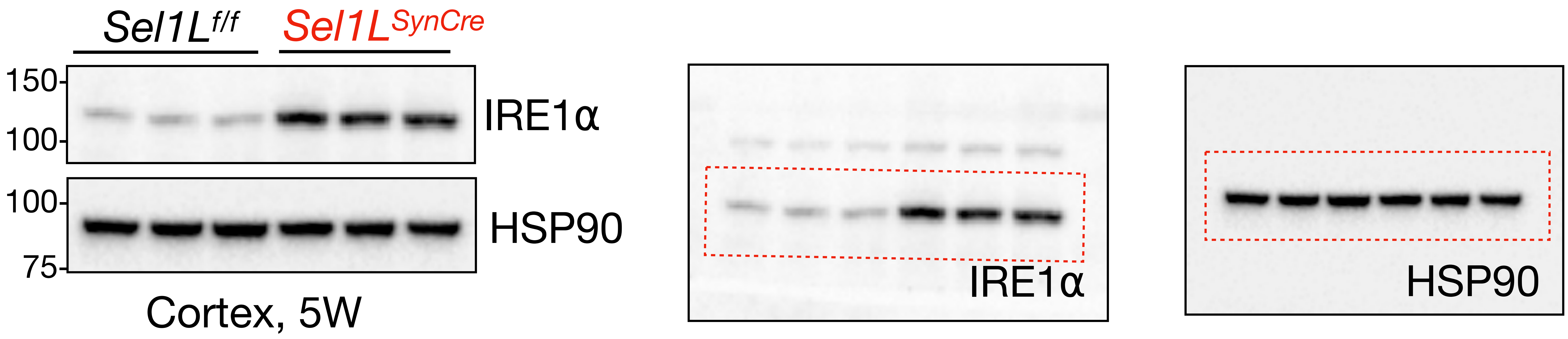

E

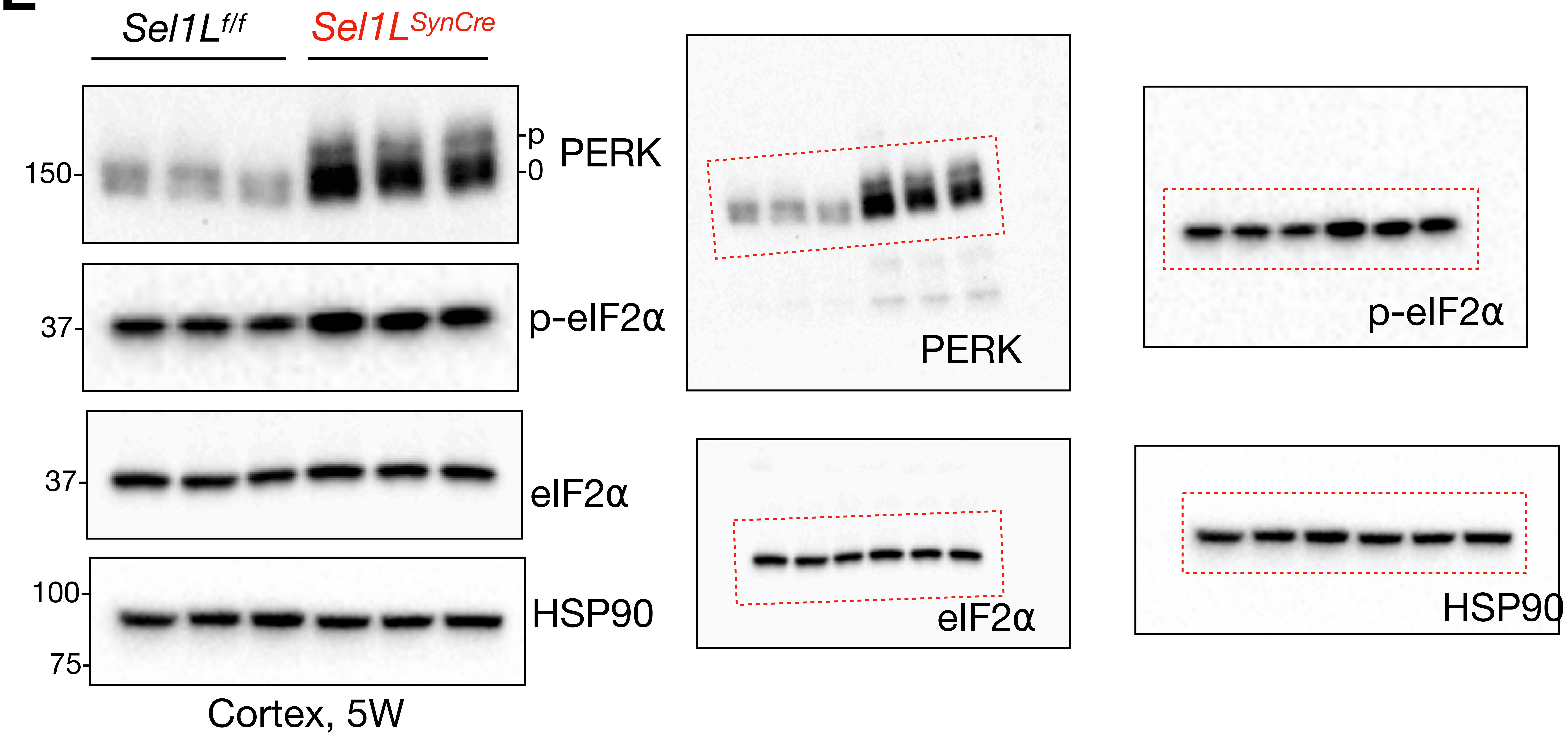

Figure 6C

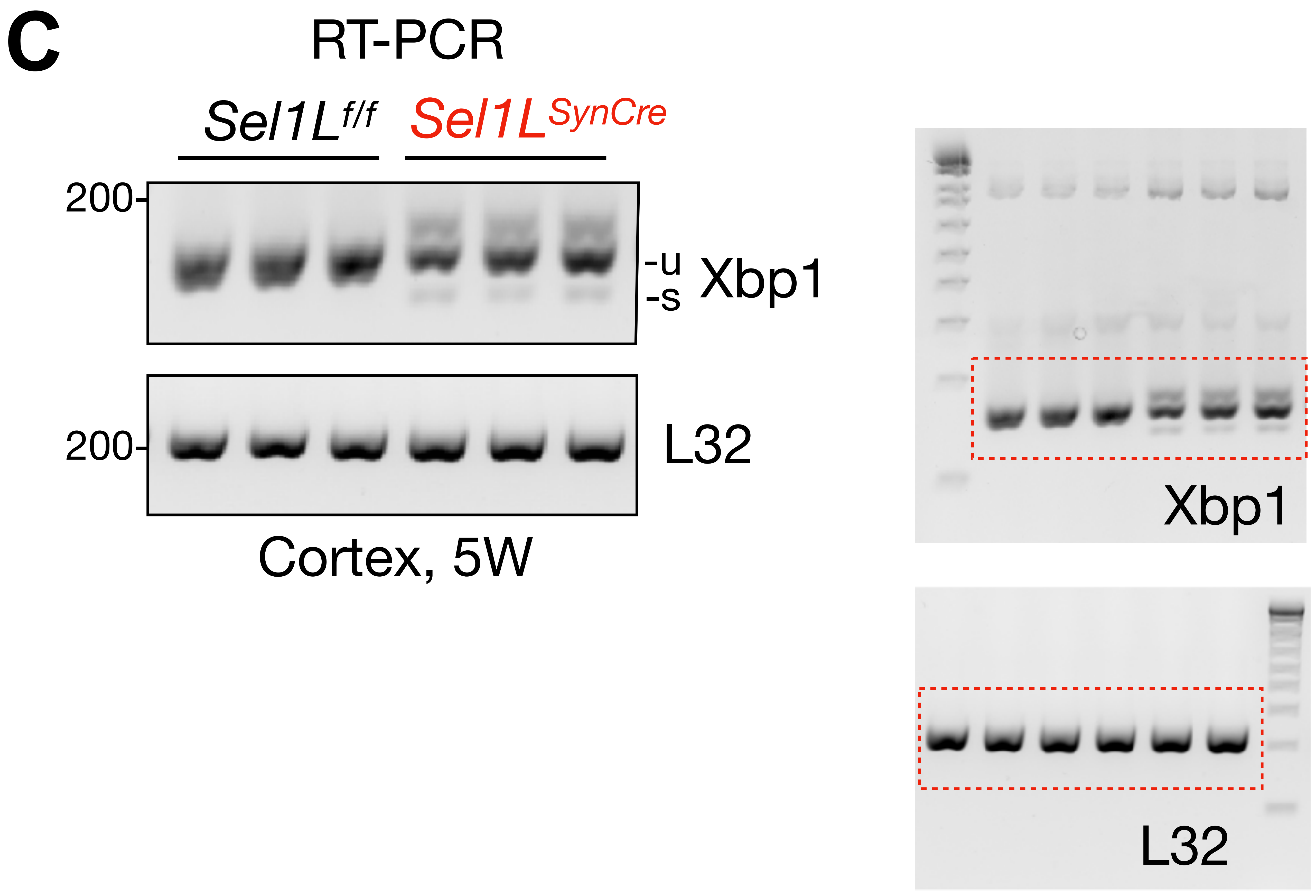
C
RT-PCR
Sel1L f/f
Sel1L SynCre
200-
-u
-s
Xbp1
200-
L32
Cortex, 5W
Xbp1
L32

Figure 8A

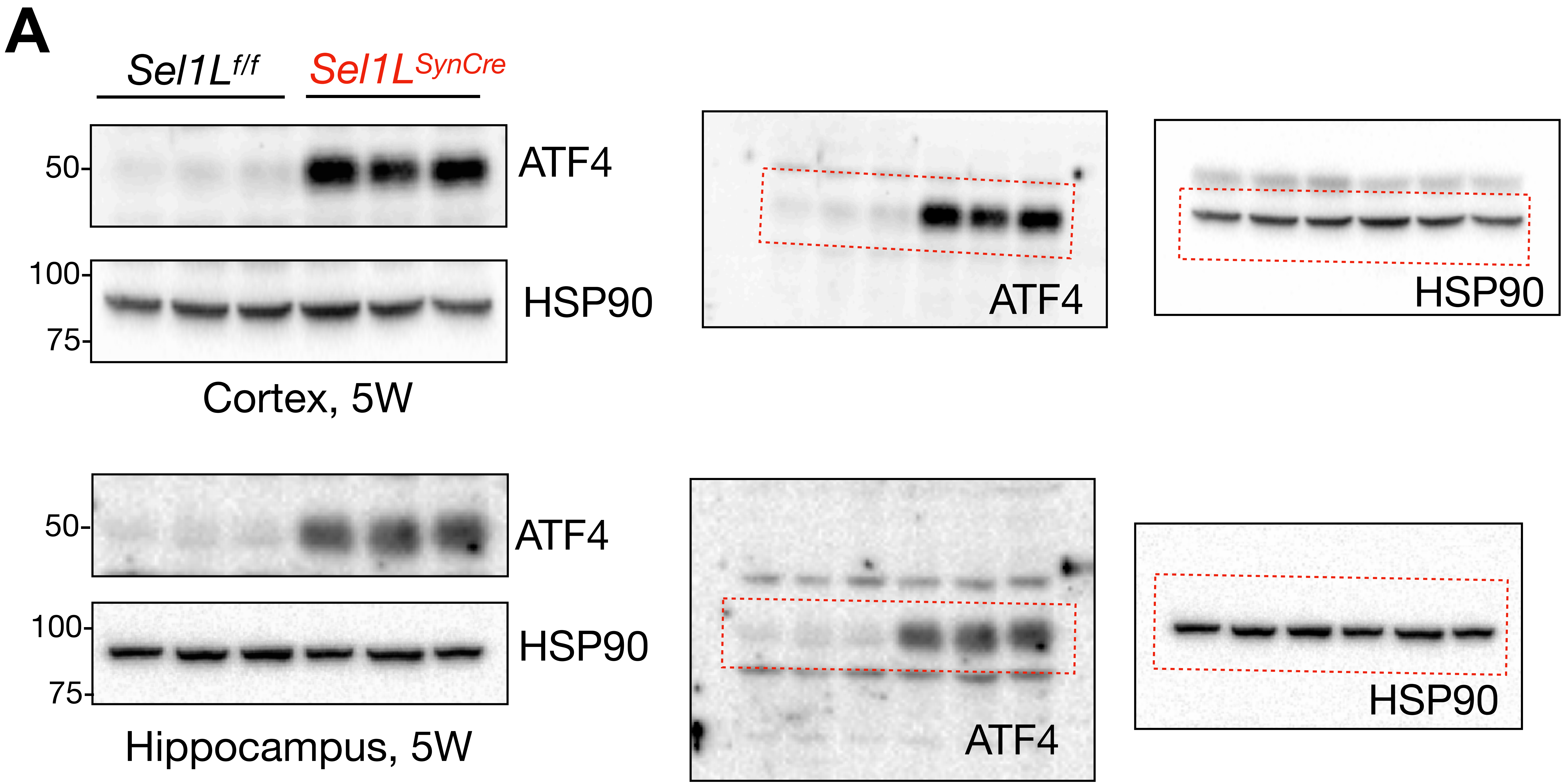
A
Sel1L f/f
Sel1L SynCre
50-
ATF4
100-
HSP90
75-
Cortex, 5W
ATF4
HSP90
50-
ATF4
100-
HSP90
75-
Hippocampus, 5W
ATF4
HSP90

Supplementary Figure 2A

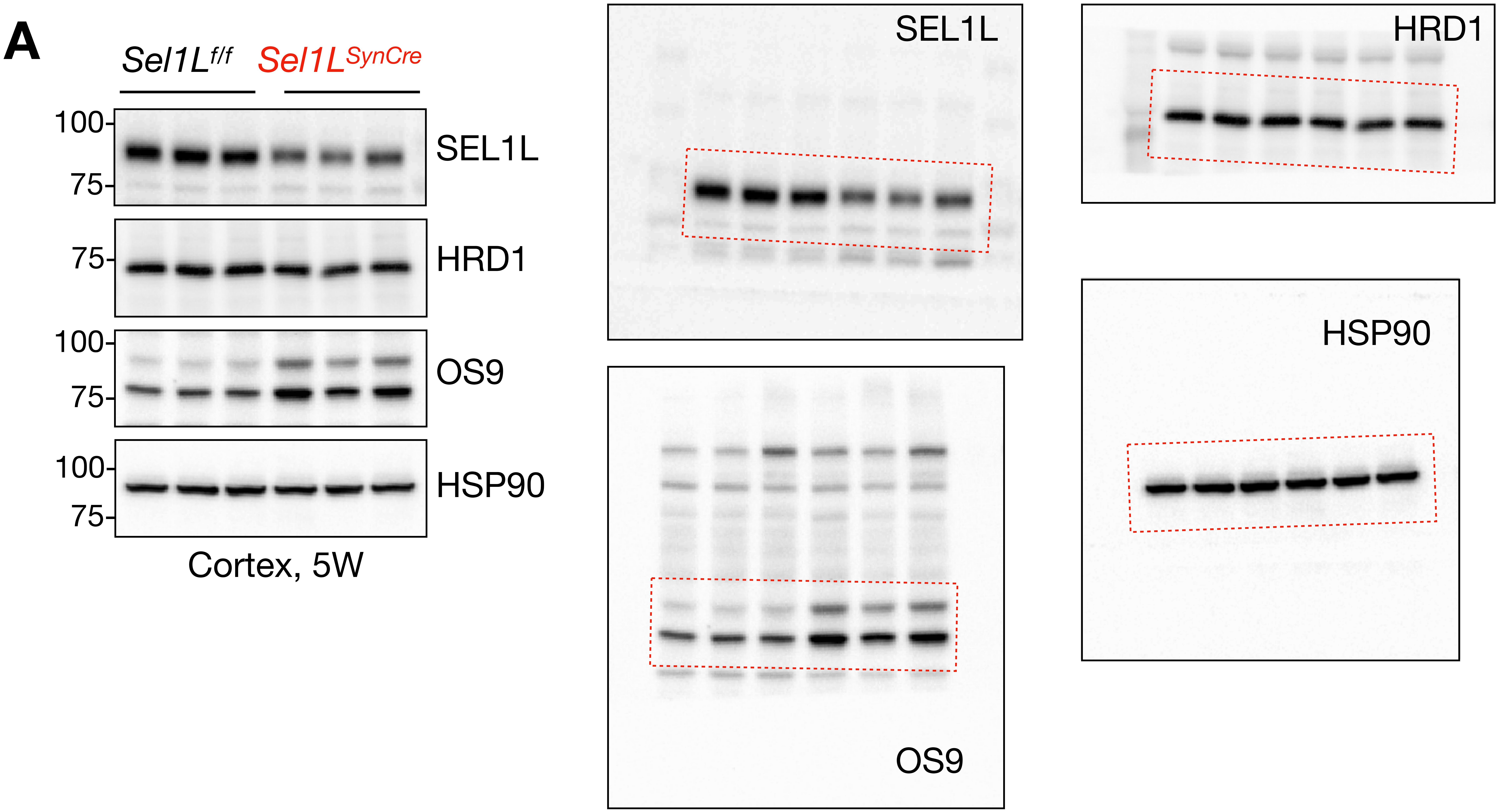

Supplementary Figure 2C

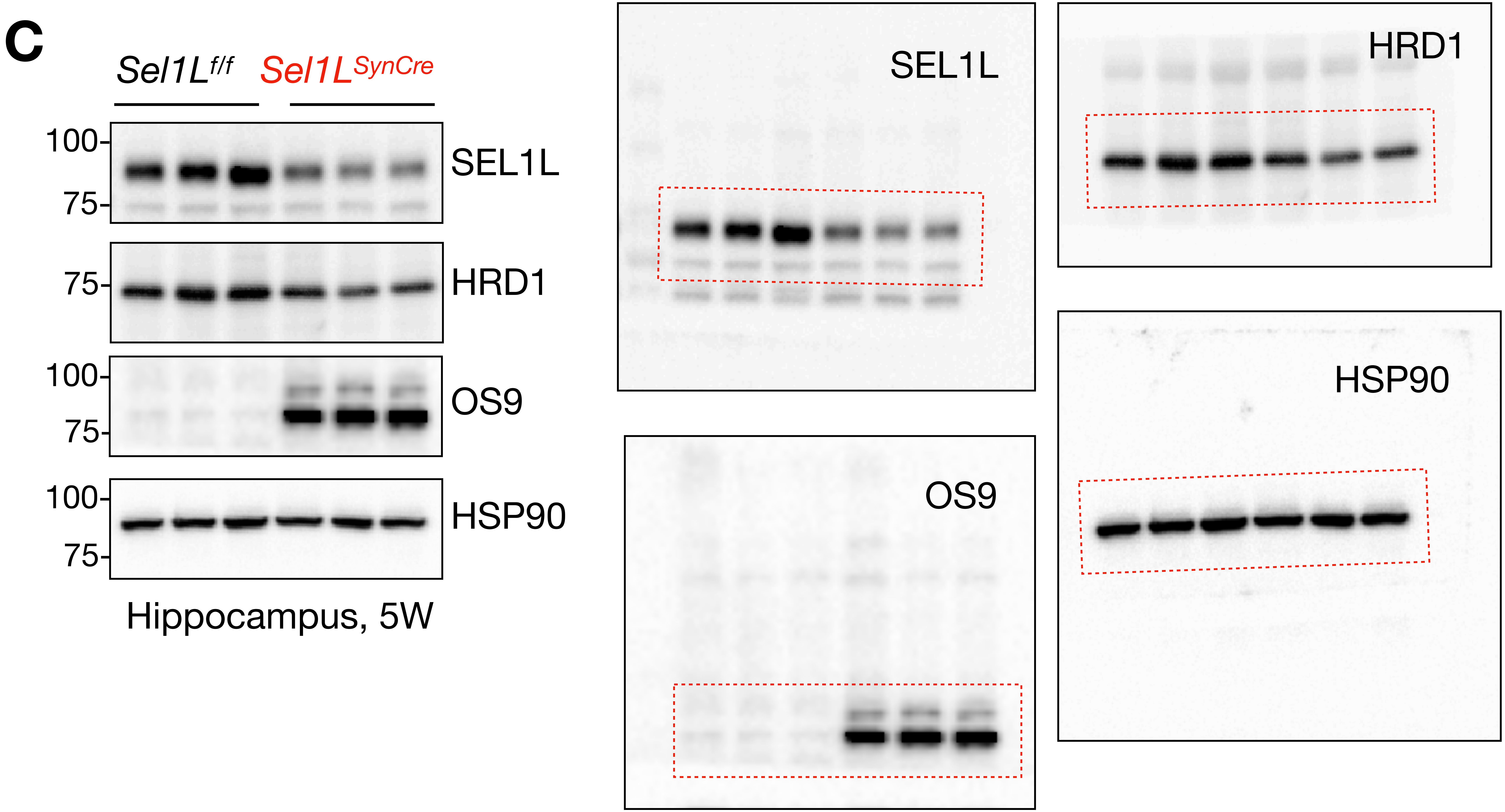

Supplementary Figure 4A

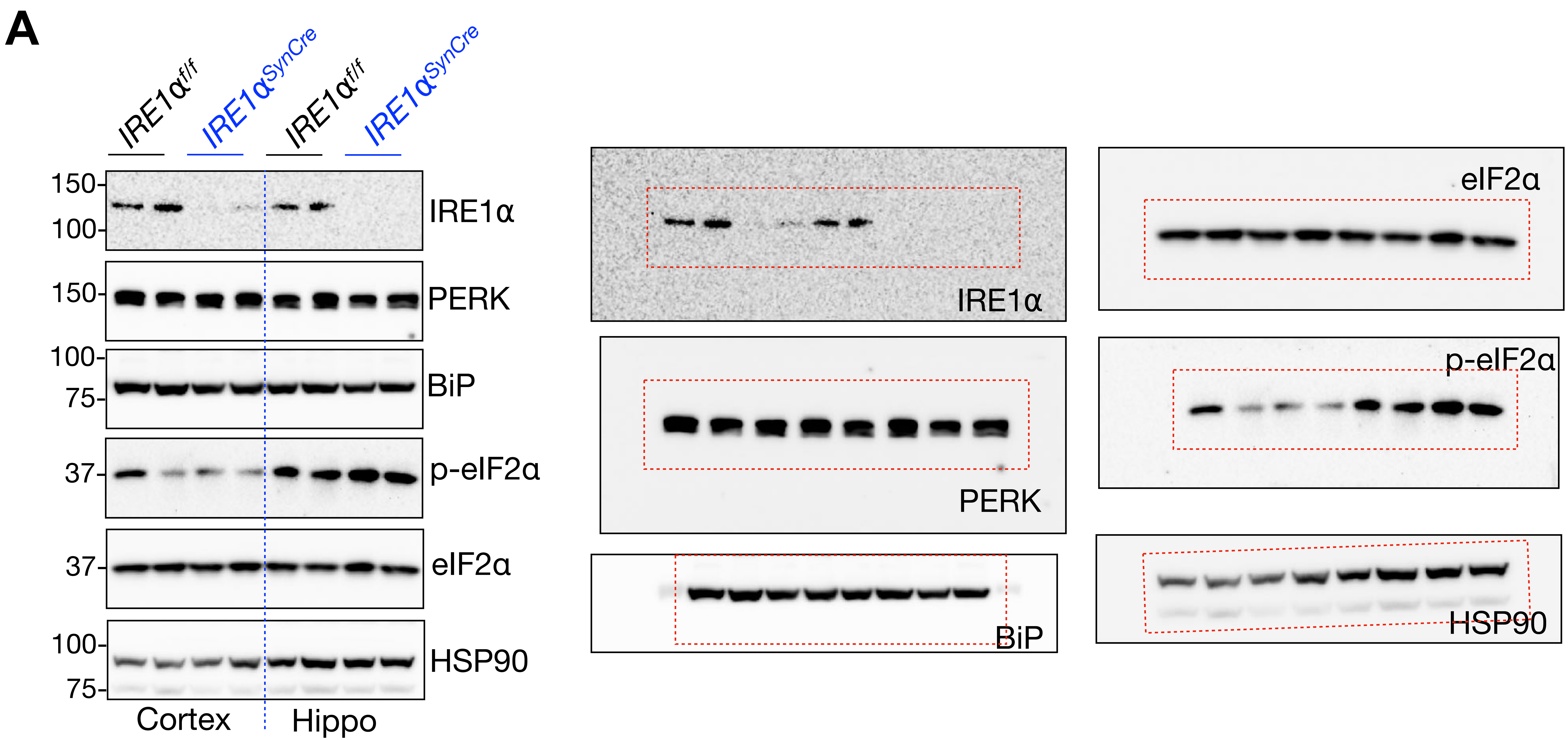

Supplementary Figure 5E

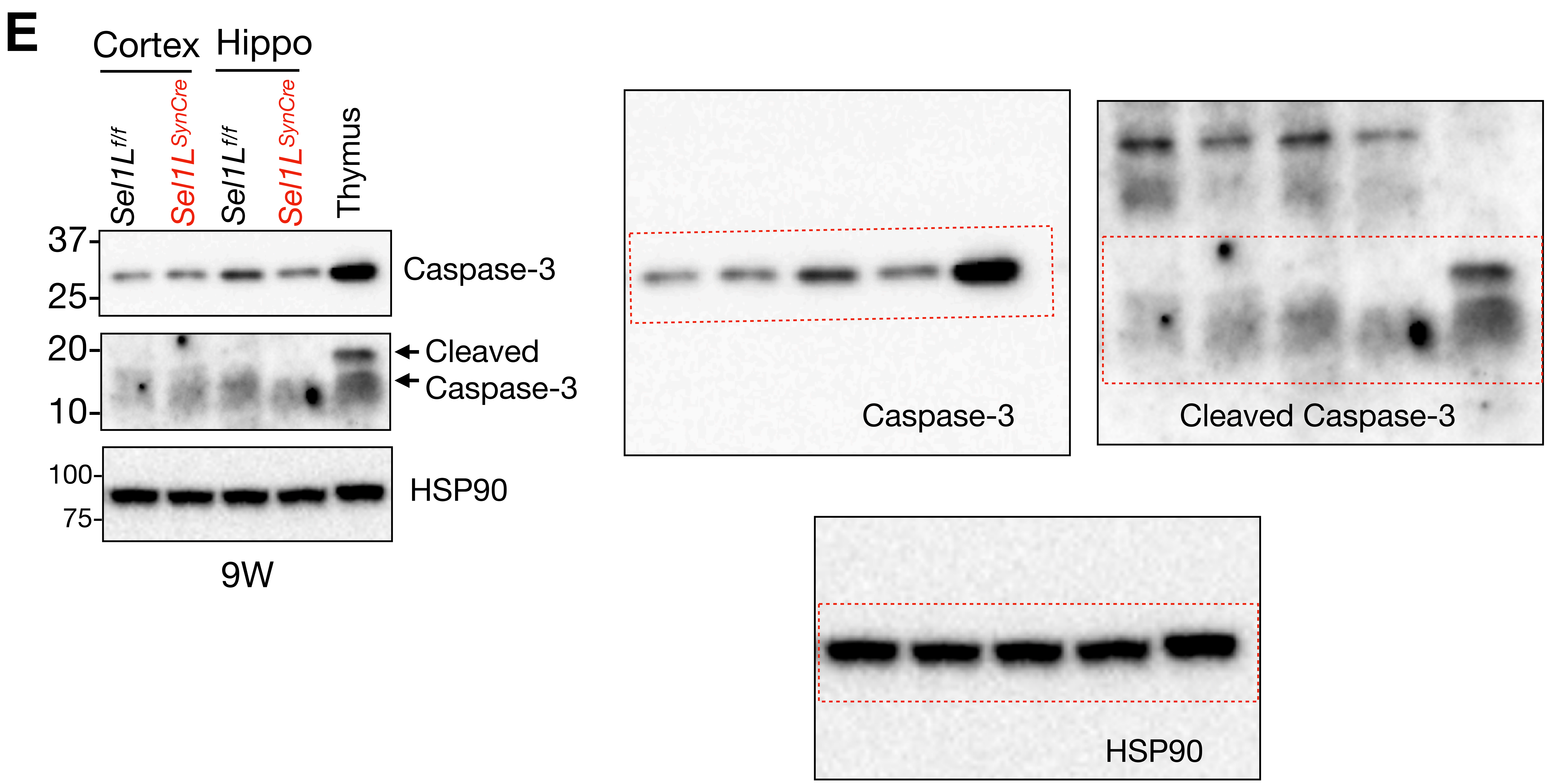

Supplementary Figure 9A

A

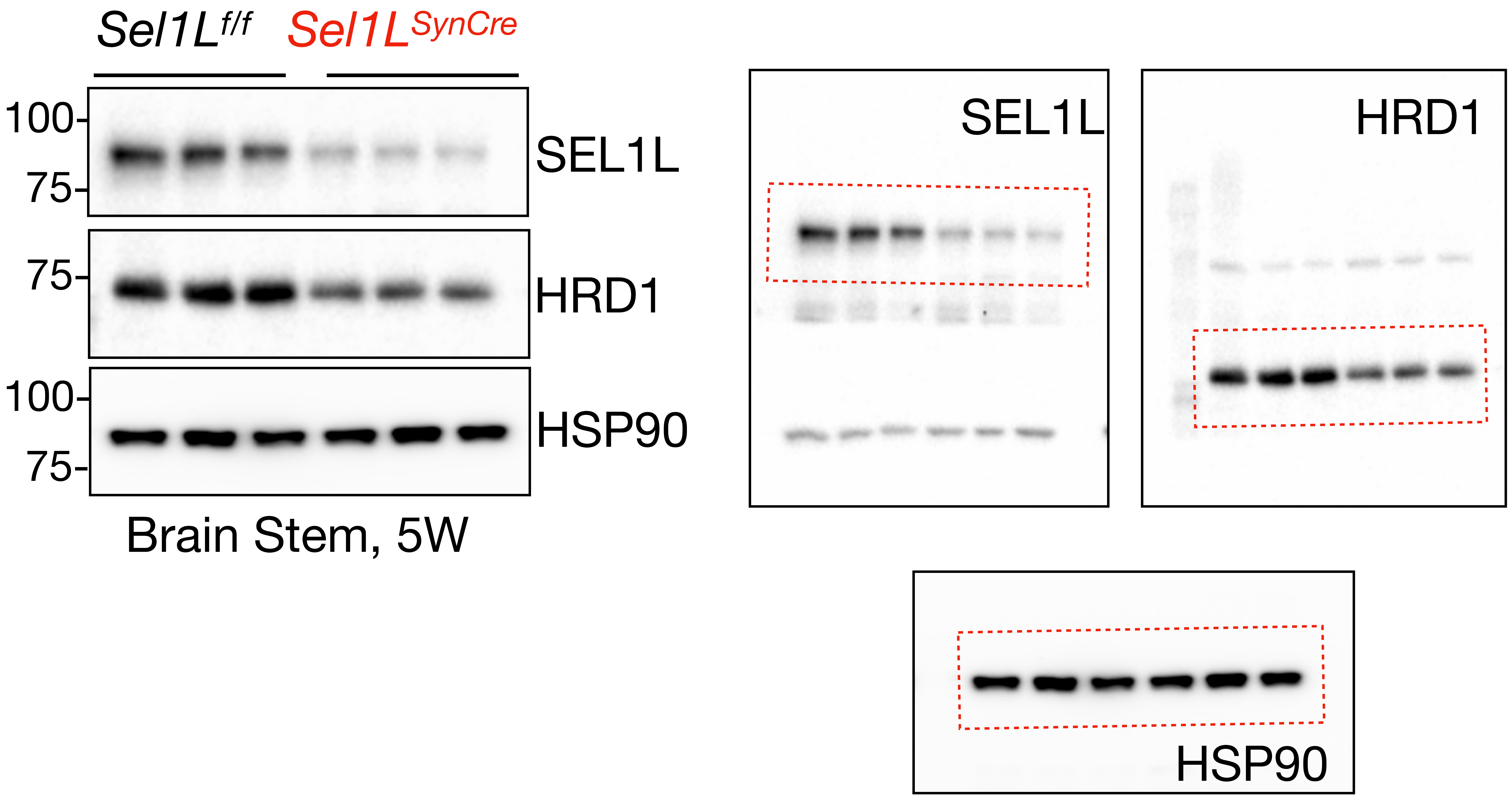

Supplement: Unedited blot and gel images [file jci-136-196819-s079.pdf]
